# Printable carbon nanotube superplastics for thermal management

**DOI:** 10.1093/nsr/nwag189

**Published:** 2026-03-27

**Authors:** Li Chen, Xiangzheng Jia, Huili Fu, Zhengpeng Yang, Yongyi Zhang, Dapeng Liu, Xinyin Yang, Jiahao Zhan, Jiangtao Di, Liming Zhao, Yufang Cao, Kunjie Wu, Zhenzhong Yong, Shaoli Fang, Muqiang Jian, Enlai Gao, Jin Zhang, Qingwen Li, Ray H Baughman

**Affiliations:** School of Nano-Tech and Nano-Bionics, University of Science and Technology of China, Hefei 230026, China; Key Laboratory of Multifunctional Nanomaterials and Smart Systems, Suzhou Institute of Nano-Tech and Nano-Bionics, Chinese Academy of Sciences, Suzhou 215123, China; Department of Engineering Mechanics, School of Civil Engineering, Wuhan University, Wuhan 430072, China; Key Laboratory of Multifunctional Nanomaterials and Smart Systems, Suzhou Institute of Nano-Tech and Nano-Bionics, Chinese Academy of Sciences, Suzhou 215123, China; Henan Key Laboratory of Materials on Deep-Earth Engineering, School of Materials Science and Engineering, Henan Polytechnic University, Jiaozuo 454003, China; School of Nano-Tech and Nano-Bionics, University of Science and Technology of China, Hefei 230026, China; Key Laboratory of Multifunctional Nanomaterials and Smart Systems, Suzhou Institute of Nano-Tech and Nano-Bionics, Chinese Academy of Sciences, Suzhou 215123, China; Division of Nanomaterials and Jiangxi Key Lab of Carbonene Materials, Jiangxi Institute of Nanotechnology, Nanchang 330200, China; Key Laboratory of Multifunctional Nanomaterials and Smart Systems, Suzhou Institute of Nano-Tech and Nano-Bionics, Chinese Academy of Sciences, Suzhou 215123, China; Henan Key Laboratory of Materials on Deep-Earth Engineering, School of Materials Science and Engineering, Henan Polytechnic University, Jiaozuo 454003, China; Beijing Science and Engineering Center for Nanocarbons, Beijing National Laboratory for Molecular Sciences, College of Chemistry and Molecular Engineering, Peking University, Beijing 100871, China; Beijing Graphene Institute (BGI), Beijing 100095, China; School of Nano-Tech and Nano-Bionics, University of Science and Technology of China, Hefei 230026, China; Key Laboratory of Multifunctional Nanomaterials and Smart Systems, Suzhou Institute of Nano-Tech and Nano-Bionics, Chinese Academy of Sciences, Suzhou 215123, China; School of Nano-Tech and Nano-Bionics, University of Science and Technology of China, Hefei 230026, China; Key Laboratory of Multifunctional Nanomaterials and Smart Systems, Suzhou Institute of Nano-Tech and Nano-Bionics, Chinese Academy of Sciences, Suzhou 215123, China; Key Laboratory of Multifunctional Nanomaterials and Smart Systems, Suzhou Institute of Nano-Tech and Nano-Bionics, Chinese Academy of Sciences, Suzhou 215123, China; Key Laboratory of Multifunctional Nanomaterials and Smart Systems, Suzhou Institute of Nano-Tech and Nano-Bionics, Chinese Academy of Sciences, Suzhou 215123, China; Key Laboratory of Multifunctional Nanomaterials and Smart Systems, Suzhou Institute of Nano-Tech and Nano-Bionics, Chinese Academy of Sciences, Suzhou 215123, China; Division of Nanomaterials and Jiangxi Key Lab of Carbonene Materials, Jiangxi Institute of Nanotechnology, Nanchang 330200, China; AlanG. MacDiarmid NanoTech Institute, University of Texas at Dallas, Richardson, TX 75080, USA; Beijing Graphene Institute (BGI), Beijing 100095, China; Department of Engineering Mechanics, School of Civil Engineering, Wuhan University, Wuhan 430072, China; Beijing Science and Engineering Center for Nanocarbons, Beijing National Laboratory for Molecular Sciences, College of Chemistry and Molecular Engineering, Peking University, Beijing 100871, China; Beijing Graphene Institute (BGI), Beijing 100095, China; School of Materials Science and Engineering, Peking University, Beijing 100871, China; School of Nano-Tech and Nano-Bionics, University of Science and Technology of China, Hefei 230026, China; Key Laboratory of Multifunctional Nanomaterials and Smart Systems, Suzhou Institute of Nano-Tech and Nano-Bionics, Chinese Academy of Sciences, Suzhou 215123, China; AlanG. MacDiarmid NanoTech Institute, University of Texas at Dallas, Richardson, TX 75080, USA

**Keywords:** nanotube-polymer fusion, hot working, high performance, processability, thermal management

## Abstract

While plastics are integral to modern life, their limitations in mechanical, thermal, and electrical properties hinder their use in advanced applications, particularly thermal management. We have developed a scalable *in-situ* nanotube-polymer fusion and hot-working process for mass-producing carbon nanotube superplastics (CNTSPs) designed to overcome these limitations. By integrating up to 59 wt% CNTs and inducing spontaneous fusion with polymer molecules while retaining the original long CNT length, we achieve enhanced CNT alignment and packing density. This unique structure yields CNTSPs with exceptional thermal performance: a high thermal conductivity (143 ± 5.8 W m^–1^ K^–1^) and a highly directional heat transfer, evidenced by a thermal conductivity anisotropy ratio of ∼123, which is crucial for efficient electronic device cooling when the heat sink is located at opposite ends of the device. Additionally, these CNTSPs exhibit high mechanical strength (663 ± 18 MPa) and electrical conductivity (8.6 × 10^4^ S m^–1^). We demonstrate the ability to shape CNTSPs into various objects by three-dimensional printing and hot pressing. This progression provides a scalable approach for mass production of superplastics combining the core advantages of CNTs with the processability of conventional plastics.

## Teaser

The mass production of printable carbon nanotube superplastics is described which provide the thermal conductivities and their high anisotropy ratio that are especially useful for device cooling.

## INTRODUCTION

Plastics have widespread applications in modern life due to their excellent processability, as well as other properties and aspects, such as light weight and low cost [1–3]. However, fabricating plastics that combine superb mechanical, electrical, and thermal properties in desired directions, defined as superplastics, remains a challenge, limiting their cutting-edge applications in advanced materials and devices [4–6]. For example, conducting polymers such as polyacetylene, polyaniline, and polypyrrole exhibit high electrical conductivity but suffer from low thermal conductivity (∼0.1 W m^–1^ K^–1^) [7,8]. Carbon nanotubes (CNTs), with ultrahigh mechanical, thermal, and electrical properties [9–12], are ideal candidates for enhancing the performance of plastics [13–17], especially when they are highly oriented in polymers.

Efficient thermal management, particularly using thin films with a high thermal conductivity anisotropy ratio (*ρ* = *κ*_max_/*κ*_min_, where *κ*_max_ and *κ*_min_ are the thermal conductivities along the high and low thermal conductivity sheet directions, respectively), is critical for integrated electronic devices in which the heat sink is in one sheet direction [18,19]. Thin films with a high *ρ* enable efficient heat dissipation, preventing overheating while maximizing the performance of electronic components in compact device architectures [19]. Combining the core advantages of CNTs and plastics is a promising approach for creating advanced materials with exceptional properties for thermal management solutions and other high-performance applications.

To this end, long-term efforts have been devoted to fabricating CNT-reinforced polymers. The uniform dispersion of CNTs, along with the formation of highly aligned and high-loading CNT networks and the retention of their original long length, is critical for achieving high mechanical performance combined with outstanding electrical and thermal properties [20,21]. Previous attempts usually involve incorporating CNT powders into polymer matrices [22–25]. While this method is straightforward, scalable, and cost-effective, it faces significant limitations, such as low CNT content, short CNT lengths, uneven filler dispersion, and random orientation. Achieving a uniform, highly aligned, and high-loading CNT network with long nanotubes in polymers to create effective pathways for load transfer, as well as heat and electron transport, remains a significant challenge, hindering performance improvements.

Polymers have been introduced into loose or dense CNT assemblies, including foams [26], films [27], fibers [28,29], and vertically aligned arrays [30,31]. However, the inherently nonpolar nature of CNT surfaces results in poor wettability with inorganic solvents such as water, making it difficult to achieve high CNT content. Consequently, these composites either contain low concentrations of CNTs, which limits the enhancements of mechanical, electrical, and thermal properties, or lack the processability of traditional polymers. The emergence of floating catalyst chemical vapor deposition (FCCVD) technology has enabled the continuous and scalable production of CNTs [32]. CNT aerogels prepared using this method can be directly deposited onto commercial polymer substrates, such as transparent tapes [33], providing significant advantages in controlling CNT loading and achieving large-area scalable production. However, compressing CNT aerogels onto polymer substrates inherently reduces porosity and restricts CNT-polymer interpenetration, limiting the formation of an integrated composite structure. In addition, many commonly used engineering plastics, such as polyamide 6 (PA6), polyacrylonitrile (PAN), polyetherketoneketone (PEKK), polycarbonate (PC), and polyvinyl pyrrolidone (PVP), are primarily soluble in organic acids, which further complicates infiltration-based approaches and inhibits effective CNT-polymer integration. This significantly restricts the scalability of CNT-reinforced polymers. As a result, fabricating carbon nanotube superplastics (CNTSPs) that combine exceptional thermal conductivity and electrical conductivity in appropriate directions, as well as plastic processing capability, remains a major challenge.

**Figure 1.**
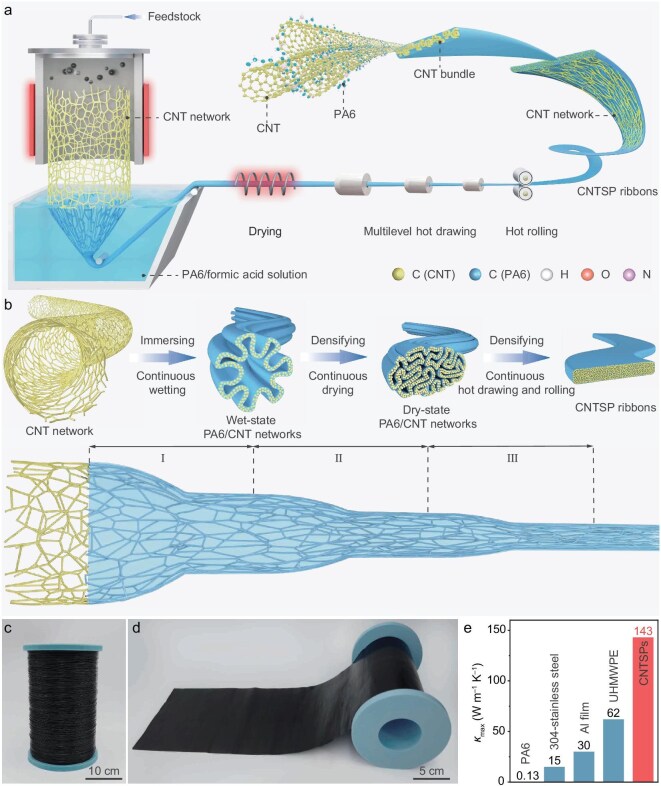
Preparation of CNTSPs. (a) Schematic diagram of the fabrication process using the NP-FHW process. (b) Schematic diagram of structural evolution of carbon nanotube (CNT) superplastic (CNTSP) ribbons. (c, d) Photographs of various shapes of CNTSPs, including (c) a 300-m-long fiber, and (d) a plastic film that is 17-cm-wide and 500-cm-long. (e) Comparison of the thermal conductivities along the high thermal conductivity directions (*κ*_max_) of CNTSPs, 304-stainless steel, Al film, ultrahigh-molecular-weight polyethylene (UHMWPE) ultradrawn film, and polyamide 6 (PA6).

We here present a scalable *in-situ* nanotube-polymer fusion and hot-working (NP-FHW) process for fabricating CNTSP ribbons with highly aligned, high-loading CNT networks, while preserving the original long CNT length (Fig. 1a). The NP-FHW process begins with the fabrication of polymer composite yarn ribbons by infiltrating the as-grown CNT networks, produced via FCCVD, into a PA6/formic acid solution. During this stage, the as-grown CNT networks spontaneously capture and fuse with polymer molecules, forming wet-state polymer/CNT networks. After thermal evaporation of the solvent, the resulting dry-state composites are subjected to a multilevel hot drawing and hot rolling process to fabricate CNTSPs using customized industrial equipment (Fig. S1b). The NP-FHW process preserves the high-loading, as-grown CNT networks while significantly enhancing CNT orientation. The combined effects of improved alignment, increased compactness, and retained original long CNT length result in improved mechanical, thermal, and electrical properties along the drawing direction (up to 663 ± 18 MPa, 143 ± 5.8 W m^–1^ K^–1^, and 8.6 × 10^4^ S m^–1^, respectively). Notably, the CNTSPs exhibit highly anisotropic thermal conductivity, with an anisotropy ratio up to 123 for orthogonal directions, making them highly promising for thermal management in electronic applications. Furthermore, we demonstrate the ability to shape CNTSPs into diverse objects via three-dimensional (3D) printing and hot pressing, offering versatility in design and functionality for advanced applications.

## RESULTS AND DISCUSSION

### Fabrication and structure of CNTSPs

Achieving high mechanical performance along with outstanding electrical and thermal properties requires the uniform dispersion of CNTs, the formation of highly aligned and high-loading CNT networks, and the retention of their original long CNT length. To this end, we developed the NP-FHW process for integrating FCCVD-grown CNT networks into polymers for the production of CNTSPs (Fig. S1a). We provided structural evolution during the fabrication of CNTSPs, as illustrated in Fig. 1b. First, a CNT network was grown using FCCVD and continuously drawn from the reactor. The length of the CNTs within the network is ∼100 µm [34]. This CNT network exhibits a loose networked structure with numerous Y-type junctions between the nanotube bundles, where the spacing between these junctions exceeds 1 μm (Fig. S2a and b). Next, the loose CNT network was *in-situ* immersed into a PA6/formic acid solution. Upon immersion, PA6 molecules were captured by the CNT network, forming a wet-state PA6/CNT network (Fig. S2c). Contact angle experiments and atomistic simulations indicate a high affinity between the PA6/formic acid solution and the loose CNT network, with PA6 preferentially fusing with the CNT network (Fig. S3, see Note S1 for details). To further investigate the wet-state PA6/CNT network, we conducted SEM and EDS mapping on freezing-dried samples. The results revealed that loose CNT networks contracted and PA6 was adsorbed onto CNT surfaces (Fig. S4). The thickness of PA6/CNT network was ∼50 nm (Fig. S2d), indicating nanoscale fusion between the polymer and CNT.

Next, the wet-state PA6/CNT network was densified by thermal evaporation of solvent. This resulted in a dry-state PA6/CNT network with a cross-section area that was significantly smaller than the wet-state structure (Fig. S2e), with both the CNTs and polymers uniformly distributed within the composite structure (Fig. S2f). However, the densification and alignment of CNTs within the dry-state structure were not yet sufficient (Figs S5 and S6a). To address this issue, multilevel hot drawing and mechanical rolling were introduced, leading to the formation of highly aligned and highly CNT-loaded CNTSPs, while retaining the original long CNT length (Figs S2g, S7, S8 and see Note S2 for details). The cross-sectional and longitudinal sectional SEM images of the CNTSPs show that the CNT network from as-grown FCCVD is well preserved, densified, and aligned (Figs S2h and S6b). Finally, the CNTSP ribbons can be processed into various configurations, such as fibers and films, using customized industrial equipment (Fig. 1c, d and Fig. S1b). Benefiting from the well-preserved CNT network, the thermal conductivity along the CNT orientation direction (high thermal conductivity direction) is over three orders-of-magnitude higher than for typical polymers (0.1 W m^–1^ K^–1^) [7] and comparable to metals, such as 304-stainless steel (∼15 W m^−1^ K^−1^) and Al–Si alloys (∼121 W m^–1^ K^–1^) (Fig. 1e and Table S1) [35,36]. Following the described procedures and using different concentrations of PA6 polymer in formic solutions, we fabricated CNTSPs with varying mass fractions of CNTs (from 17 to 74 wt%). The dispersion and mass fractions of CNT were characterized by SEM and thermogravimetric analysis (TGA) measurements (Figs S9 and S10). It was observed that when the CNT mass fraction exceeds 59 wt%, the processability significantly decreases (Fig. S11 and see Note S3 for details). Hence, CNTSPs with a mass fraction of CNTs below 59 wt% are the focus of the following discussion. For comparison, the corresponding volume fractions of CNTs in the CNTSPs are provided in Table S2.

**Figure 2.**
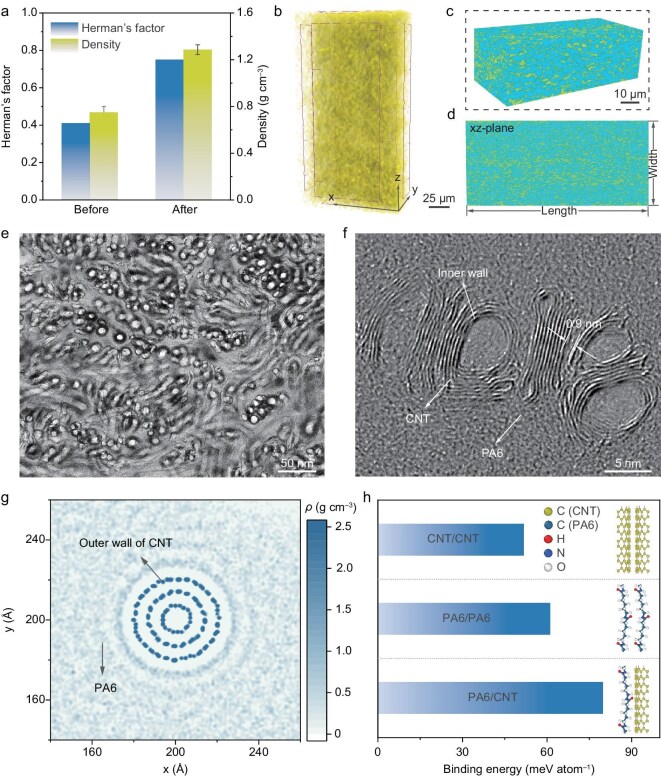
Structures of CNTSPs. (a) Comparison of density and the alignment before and after hot drawing and rolling. (b) Three-dimensional (3D) CNT networks and (c) CNTSPs reconstructed by nanoscale X-ray computed tomography. (d) An xz-plane representation of the scanning results in (b). The yellow regions in (b–d) represent CNTs and the blue regions represent PA6. (e) Low-resolution and (f) high-resolution TEM images of a CNTSP sheet plane. (g) Theoretically calculated mass density profile of a CNT surrounded by PA6 chains. (h) Theoretically calculated binding energy of different interfaces.

We further examined the CNTSPs to investigate the distribution of CNTs, densification, alignment, and interfacial structures. First, we conducted density and alignment measurements using wide-angle X-ray scattering (WAXS) analyses (Fig. S12). The results indicate that the density and Herman’s orientation factor (*f*) of CNTSPs, before and after the multilevel hot drawing and mechanical rolling, increased from 0.75 to 1.33 g cm^–3^ and from 0.41 to 0.75, respectively (Fig. 2a). Furthermore, the orientation of the CNT network within the CNTSPs was quantified using polarized Raman spectroscopy. The ratio of the G-band intensities measured parallel and perpendicular to the drawing direction (*I*_G∥_/*I*_G⊥_) increased significantly from 1.25 to 4.18 after the hot drawing and rolling process (Fig. S13). This nearly fourfold increase directly demonstrates a transition from a semi-isotropic state to a highly oriented configuration throughout the bulk material. These spectroscopic results are highly consistent with the orientation factors derived from WAXS (Fig. S12). This suggests that the densification and alignment of CNTSPs were significantly enhanced. Next, the 3D microstructure of CNTSPs was characterized using nanoscale X-ray computed tomography (nano-CT; Fig. 2b–d). Nano-CT results demonstrate that as-grown CNT networks were well preserved and uniformly distributed, with the voids within CNTSPs largely eliminated (Fig. S14 and Tables S3 and S4). To explore the interfacial structure between CNTs and PA6, we conducted transmission electron microscope (TEM) and atomistic simulations. TEM images obtained from focused ion beam (FIB) cutting of CNTSPs (Fig. 2e) revealed that the oriented CNT networks were well fused with PA6. The presence of PA6 was confirmed by comparing TEM images of a pure CNT ribbon and a CNTSP ribbon (Fig. S15). High-resolution TEM images show that PA6 can infiltrate the nano-channels between CNTs (Fig. 2f), indicating that PA6 is adsorbed onto CNT surfaces. These experimental findings were supported by our atomistic simulations, which demonstrated that the calculated binding energy between CNT and PA6 (79.9 meV/atom) is significantly higher than the binding energy between CNT and CNT (51.7 meV/atom) or PA6 and PA6 (61.3 meV/atom). Thus, the PA6 molecules are strongly adsorbed onto CNT surfaces (Fig. 2g, h and Figs S15, S16). The binding energy and adsorption process were calculated using density functional theory-based tight-binding and molecular dynamics simulations.

### Mechanical, thermal, and electrical performance of CNTSPs

**Figure 3.**
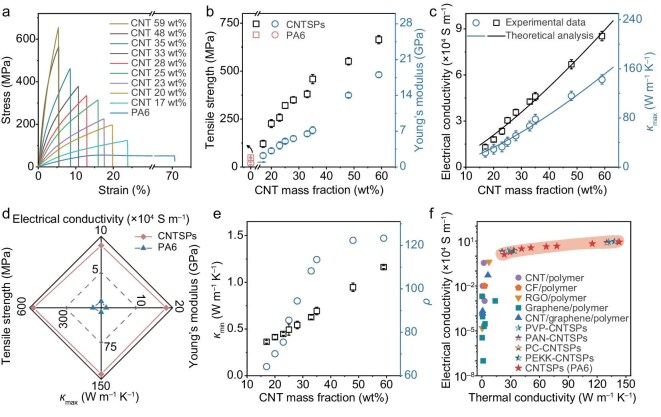
Performance of CNTSPs. (a) Representative tensile stress–strain curves for CNTSPs containing CNT mass fractions from 0 to 59 wt%. (b) Tensile strength and Young’s modulus of PA6 and CNTSPs with different CNT mass fractions. (c) Experimental and theoretical analyses of electrical conductivity in the CNT orientation direction and *κ*_max_ of CNTSPs with different CNT mass fractions. (d) Radar chart of properties of CNTSPs and PA6. (e) Thermal conductivity along a low thermal conductivity direction (*κ*_min_) and thermal conductivity anisotropy ratio (*ρ*) vs. the CNT mass fraction for CNTSPs. (f) Electrical and thermal conductivities of CNTSPs compared to previously reported nanocarbon-based plastics, highlighting the significantly enhanced multifunctional performance of our CNTSPs.

The NP-FHW process significantly enhances the mechanical, thermal, and electrical properties of CNTSPs. To quantify these improvements, we performed tensile tests on CNTSPs with CNT mass fractions between 17 and 59 wt% (Fig. S17 and Table S5). Typical tensile stress–strain curves are shown in Fig. 3a, from which Young’s modulus and tensile strength were derived (Fig. 3b). These results show that the Young’s modulus and tensile strength dramatically increased from 0.7 ± 0.2 GPa and 59 ± 11 MPa for pure PA6 to 18.1 ± 0.6 GPa and 663 ± 18 MPa for CNTSPs, respectively, demonstrating a significant improvement in mechanical performance. Notably, the tensile strength of CNTSPs in the CNT orientation direction is comparable to metals, such as Al-alloy (∼400 MPa) and Mg-alloy (∼280 MPa) [37,38]. In addition to tensile performance, we also investigated the resistance to stress relaxation of CNTSPs using stress-relaxation experiments (Fig. S18). The CNTSPs exhibit a significantly higher resistance to stress relaxation compared to pure PA6. This enhancement is attributed to the strong interfacial interactions between the CNT and PA6, which can be supported by atomistic simulations of binding energy (Fig. 2h).

Then, we evaluated the electrical performance of CNTSPs. Remarkably, along the CNT alignment direction, the electrical conductivity increased nearly 7-fold, from 1.3 × 10^4^ to 8.6 × 10^4^ S m^–1^, as the CNT mass fraction was raised from 17 to 59 wt% (Fig. 3c). The performance stability of CNTSPs was examined by measuring the electrical conductivities of CNTSPs after they were exposed to various mechanical and thermal conditions. The electrical conductivity of CNTSPs in the CNT orientation direction was little changed during the sharp bending shown in Fig. S19a for 10 000 cycles. Also, the electrical conductivity along the CNT orientation direction changed by <5% when subjected to atmospheric air at temperatures ranging from 25 to 220°C for 10 min (Fig. S19b, see Note S4 for details). Furthermore, stability tests conducted at 220 °C for 36 h demonstrate the excellent durability of CNTSP, confirming its suitability for electronic thermal management (Fig. S19c).

We next characterized thermal conductivities of CNTSPs using a self-heating 3ω method (Fig. S20). The thermal conductivity of pure PA6 is about 0.13 W m^–1^ K^–1^. Upon incorporating highly aligned and high-loading CNT networks (59 wt%) into these plastics, the thermal conductivity of CNTSPs along the CNT orientation direction (which is the maximum thermal conductivity of CNTSPs, *κ*_max_) increased to 143 W m^–1^ K^–1^ (Fig. 3c). These results demonstrate the effective enhancement of properties obtained by incorporating aligned long CNTs (Fig. 3d, Fig. S21, and Table S5), with the thermal conductivity of CNTSPs being over three orders-of-magnitude higher than typical polymers (∼0.1 W m^–1^ K^–1^) [7] and comparable to metals such as 304-stainless steel (∼15 W m^−1^ K^−1^) and Al–Si alloys (∼121 W m^–1^ K^–1^) [35,36].

Furthermore, due to the high orientation of CNTs and retention of the original long nanotube length for our CNTSPs, thermal conductivity is greatly enhanced along the CNT orientation direction. As a result, CNTSPs exhibit thermally anisotropic properties, with a high in-plane thermal conductivity along the CNT orientation direction (*κ*_max_, 143 W m^–1^ K^–1^) and a relatively low through-plane thermal conductivity (*κ*_min_, 1.16 W m^–1^ K^–1^) (Fig. 3e, Fig. S22, and Table S6). The *ρ* of our CNTSPs (∼123) is particularly advantageous for thermal management applications, such as in electronics. Importantly, the CNTSPs provide the highest thermal and electrical conductivities of nanocarbon-reinforced polymers found in a literature survey (Fig. 3f, Tables S7 and S8).

To understand the performance of the CNTSPs, the rule-of-mixture was used to explain the improvement in mechanical, thermal, and electrical performance of CNTSPs (Fig. 3c and Fig. S23). The classical rule-of-mixture assumes a linear relationship between the volume fraction of the reinforced filler and the overall performance of the composite [39]. The formula, *P*_c_ = *P*_1_*V*_1_ + *P*_2_(1–*V*_1_), is widely used to predict the mechanical performance of composites. However, we found that this model tends to overestimate the contribution of CNTs, particularly at lower CNT fractions. This overestimation might be because the properties of CNTs vary non-linearly with their fraction in the composite. As an alternative, we propose a modified rule-of-mixture to estimate the contribution of CNTs expressed as


\begin{eqnarray*}
{P}_{\mathrm{c}} = {P}_{{\mathrm{CNT}}}V_{{\mathrm{CNT}}}^\alpha + {P}_{{\mathrm{nyl}}}(1 - {V}_{{\mathrm{CNT}}}),
\end{eqnarray*}

where *P*_c_, *P*_CNT_, and *P*_nyl_ represent the properties of CNTSPs, CNT, and nylon (PA6), respectively, and *V*_CNT_ is the volume fraction of CNTs. This modified model agrees well with the experimental data (Fig. 3c and Table S9, see Note S5 for details), offering a more precise representation of CNTSP performance.

### The processability and applications of CNTSPs

**Figure 4.**
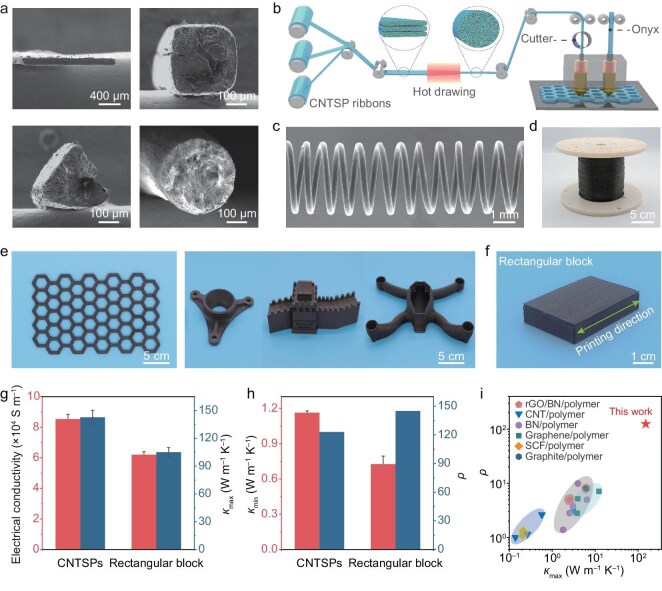
Processability of CNTSPs. (a) Heated CNTSPs can be plasticized and compression-molded into objects of various shapes, including ribbons, triangles, rectangles, and circles. (b) Schematic diagram of the 3D-printing process for the fabrication of CNTSP filaments. (c) Scanning electron microscopy (SEM) image of CNTSP filaments after a hot-drawing process. (d) Photograph of a 300-m-long roll of CNTSP filament. (e) Photographs of printed two-dimensional honeycombs and complex 3D structures. (f) Photograph of a printed rectangular block (5 cm × 3 cm × 1 cm). (g) Comparison of thermal and electrical conductivities along the printing direction between CNTSPs and the printed block. (h) Comparison of *κ*_min_ and the corresponding *ρ* between CNTSPs and the printed block. (i) Plot of *ρ* vs. *κ*_max_ for the printed block in comparison with other 3D printable plastics.

Processability is crucial for various applications, particularly for thermal management. While commercially available graphite films exhibit a high in-plane thermal conductivity and a high *ρ* (∼340), their poor processability limits their integration into electronic devices. In contrast, CNTSPs offer exceptional processability, enabling them to be shaped into diverse forms, including rectangle, triangle, circle, and various other more complicated structures (Fig. 4a and e). To further explore their versatility, we evaluated the potential of CNTSPs for 3D printing. A CNTSP filament with a diameter of 400 µm was prepared for 3D printing by hot drawing multi-filaments of CNTSPs (Fig. 4b–d). Compared with the CNTSP ribbon, the negligible variations in the electrical conductivity (8.4 × 10^4^ S m^–1^) and thermal conductivity (141 W m^–1^ K^–1^) of a single 400 µm diameter filament indicate that the hot-drawing process neither disrupted the internal CNT network nor introduced interfacial defects during the multi-filament fusion process (Fig. S24). Using a self-developed 3D printer, these CNTSP filaments were layer-by-layer printed onto the Onyx (nylon matrix composite reinforced by short carbon fiber) substrate. By alternately printing CNTSP and Onyx layers, a multilayer closely stacked monolithic architecture was regularly constructed as designed (Fig. S25a and b). The CNTSP and Onyx layers were seamlessly fused, with no noticeable gaps or holes in the interfaces, demonstrating the excellent interfacial bonding between CNTSP and Onyx layers.

The incorporation of CNTSPs significantly enhanced structural strength. Compared to 3D-printed tensile bars of Onyx and PA6, the tensile strength and Young’s modulus of 3D-printed tensile bars of CNTSPs increased with the thickness of CNTSPs printed layers, reaching a maximum of 118 ± 5 MPa and 1.75 ± 0.2 GPa, respectively (Fig. S25c and d). To evaluate the thermal and electrical conductivities of the 3D-printed CNTSP composites, we fabricated a rectangular block (5 cm × 3 cm × 1 cm, Fig. 4f). The printed block exhibits thermal and electrical conductivities of 105 W m^–1^ K^–1^ and 6.2 × 10^4^ S m^–1^ along the printing direction, respectively, retaining over 70% of the performance of CNTSP (Fig. 4g). The anisotropy ratio of the printed block reaches 145, a notable increase from the 123 observed in the base CNTSP. This enhancement is attributed to the synergistic alignment effect inherent to the extrusion-based deposition process. As the CNTSP filament is processed, the nozzle-induced shear and convergent flow provide a secondary refinement of the CNT orientation along the print path. Furthermore, the periodic interfaces formed during the layer-by-layer deposition process act as supplementary thermal barriers in the through-plane direction, moderately impeding vertical heat transport while longitudinal pathways remain highly conductive. These combined effects give rise to the high overall anisotropy ratio in the final printed structures (Fig. 4h). Notably, compared with other 3D-printable plastics, 3D-printed CNTSPs achieve more than an order-of-magnitude improvement in both *κ*_max_ and electrical conductivity, highlighting their exceptional multifunctional performance and scalability as printable, high-CNT-content materials (Fig. 4i and Table S10).

**Figure 5.**
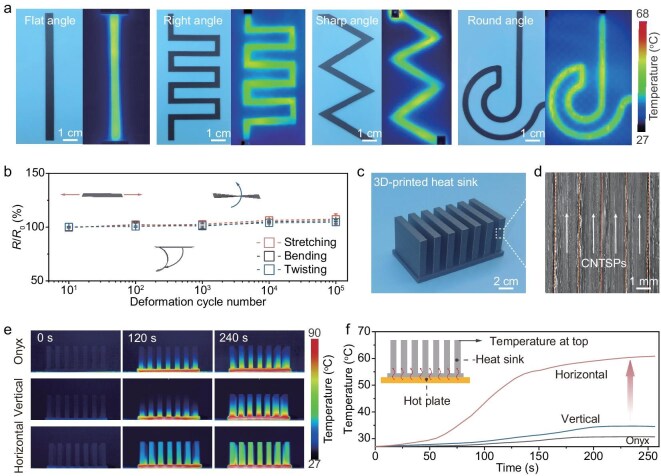
Applications of CNTSPs. (a) Photographs and infrared thermal images of various conductive paths, including circuits with straight, right-angle, triangular, and circular sides. (b) Resistance changes of flat circuits after repeated deformation: bending (curvature radius of 2 cm), twisting (twisting angle of 30°), and stretching (stretching ratio of 1%). Resistances increased by <10% after 100 000 cycles. *R*_0_ and *R* correspond to the resistances before and after deformation, respectively. (c) Photograph and (d) SEM image of the corresponding fabricated structure used for measuring CNT-direction heat transport. (e) Infrared thermal images and (f) temperature evolution curves of pure Onyx and CNTSPs heat sinks when heated by a planar heat source at 90°C over different time periods.

Using 3D printing to create customizable electrical and thermal conduction pathways is key to achieving efficient thermal management. Various printouts were successfully fabricated, with all CNTSP filaments maintaining structural integrity and continuity, even at bent or folded regions (Fig. S26, see Note S6 for details). To further assess their electrical performance, we also explored the performance of CNTSP printouts containing various conductive paths, including circuits with flat, right, sharp, and round angles. When a voltage of 10 V was applied to the ends of these printouts, Joule heat was transported smoothly along the conducting paths (Fig. 5a). To quantitatively evaluate the Joule heating performance of the 3D-printed CNTSPs, we performed a systematic analysis of components with varying path geometries. Temporal temperature responses demonstrate a rapid heating rate across all configurations, reaching steady state within seconds of energization. The flat-angle path exhibited the highest electro-thermal conversion efficiency and steady‑state temperature, a direct result of its minimized path length and lower total electrical resistance (Fig. S27a). For more complex right-angle path configurations, the material maintained excellent resistance-temperature stability over multiple heating-cooling cycles, with no observable degradation in performance (Fig. S27b). Furthermore, the long-term reliability of the customized circuitry was confirmed by continuously powering a right-angle path at 10 V for 3500 s. The surface temperature remained remarkably stable, with fluctuations restricted to ∼±1.5°C (Fig. S27c). This outstanding thermal and electrical endurance highlights the structural integrity of the CNTSP network, which remains robust even under the localized thermal stresses inherent to complex, 3D-printed conductive pathways.

Furthermore, the electrical resistances along the CNT orientation direction barely changed even after 100 000 cycles of bending, twisting, and stretching (Fig. 5b and Fig. S28). This high conductivity and durability of the CNTSP printouts can be attributed to the preserved high loading of the as-grown CNT networks with an enhanced degree of CNT orientation. These findings demonstrate the utility of CNTSPs for providing thermally conducting paths for cooling advanced functional electronic devices. For effective thermal management applications, both high *ρ* and high processability are important. Although various crystalline materials, such as graphite [40], hexagonal boron nitride [18], and MoS_2_ [19], exhibit intrinsically high *ρ*, they are often challenging to scalably stack for applications requiring a thickness >1 μm for in-plane heat transport. Also, the alternative materials provide isotropic in-plane heat transport, rather than the highly directional heat transport by CNTSPs. Utilizing 3D printing, the CNTSPs can be engineered to optimize high heat transport in only the desired direction.

We compared the performance of printouts composed of commercially available Onyx and CNTSPs aligned either parallel (vertical) or perpendicular (horizontal) to the heat flux (Fig. 5c–f and Fig. S29). The printouts were placed on a planar heat source at 90°C. The temperature distributions were monitored using a thermal imaging camera (Fig. 5e). The thermal images and temperature-time curves revealed that the CNTSP device exhibits significantly better heat transport performance along the CNT-orientation direction compared to the pure Onyx device (Fig. 5f). In contrast, the transport performance of CNTSPs perpendicular to the heat flux is quite low. The presence of long CNTs further enhances the thermal transport anisotropy of CNTSPs, optimizing their effectiveness in heat management. It is worth noting that the thickness of our 3D-printed CNTSP films can be increased up to 27 μm (Fig. S30), significantly enhancing the heat flow rate along the CNT-orientation direction, while still minimizing the heat flow rate along the perpendicular direction. This contrasts with other thin anisotropic thermal conductor films, which typically have a thickness in the nanometer range [19]. With these advantages, CNTSPs meet the critical requirements for effective thermal management in electronics: a high *κ*_max_, a high *ρ*, excellent processability, and scalability. Therefore, CNTSPs are promising candidates for thermal management solutions in electronics.

Our strategy can be extended to other plastics, enabling diverse application scenarios. To demonstrate the universality of our approach, we fabricated CNTSPs by fusing high-loading CNT networks with polymers such as PVP, PAN, PC, and PEKK (Fig. S31). Contact angle experiments and SEM images confirm the high affinity between these polymer solutions and CNT films, as well as the uniform dispersion of the as-grown CNT networks (Figs S32 and S33). Similar to PA6-CNTSPs, the mechanical, thermal, and electrical performance of PVP-, PAN-, PC-, and PEKK-CNTSPs were also significantly enhanced (Figs S34, S35, and Table S11). Thermal and electrical conductivities of all fabricated CNTSPs increased by more than two orders-of-magnitude compared to their respective pure polymers. These results indicate that the introduction of conductive CNT networks, featuring improved CNT alignment, enhanced packing density, and retention of the original long CNT length, effectively overcomes the performance bottleneck in plastic applications. Additionally, these PVP-, PAN-, PC-, and PEKK-CNTSPs also exhibited excellent processability (Fig. S36), which is crucial for potential applications, such as aircraft and spacecraft. While the processability of CNTSPs offers potential sustainability advantages through recycling and reprocessing, a comprehensive evaluation must also consider environmental impacts, such as the possible release of microplastics and embedded CNTs during degradation or throughout their lifecycle. The sustainability of CNTSPs is underpinned by the robust CNT-PA6 interfacial bonding and the thermal stability of the continuous CNT scaffold. These structural characteristics allow for multiple reprocessing cycles via 3D printing or hot-pressing; the polymer phase can be selectively melted and reshaped without disrupting the ‘locked’ CNT orientation or inducing performance degradation. This resilience enables the recycling of CNTSPs while preserving their superior anisotropic properties, providing a pathway toward sustainable high-performance composites. Future assessments will further quantify the long-term integrity of this interface and address potential risks regarding microplastic or CNT release during industrial-scale recycling.

## CONCLUSION

We have demonstrated that conveniently processed CNTSP ribbons can provide a room-temperature thermal conductivity up to 143 W m^–1^ K^–1^. Unlike conventional metals, the CNTSPs offer highly unidirectional thermal conductivity, a critical advantage for heat pipe applications in electronic device cooling. This unique property of our CNTSPs stems from the NP-FHW process, where the loose network of about 100-μm-long CNTs contracts as PA6 is captured, without losing their high CNT orientation. Contact angle experiments and atomistic simulations confirm a strong affinity between the PA6/formic acid solution and the loose CNT network, with PA6 preferentially fusing with the CNT network. While other highly thermally conductive sheets, such as graphene and h-BN, also provide high thermal conductivities, their isotropic thermal conductivities make it very difficult to achieve sheet stacks as thick as 1 μm or more—a thickness easily attained with CNTSP films or ribbons.

We also demonstrated the 3D printing of CNTSP ribbon stacks, which could be shaped into diverse architectures without losing CNT orientation, as the polymer was temporarily melted during printing. This enabled us to locally obtain high CNT orientation and correspondingly high thermal conductivity in whatever directions are desired for diverse thermal transport applications. These superplastics effectively combine the core advantages of CNTs, such as high thermal conductivity and high anisotropy, with the processability of conventional plastics, allowing scalable manufacturing and versatile shaping. This capability unlocks new possibilities for tailored thermal management solutions.

## METHODS

The detailed methods can be found in the online supplementary file.

## Supplementary Material

nwag189_Supplemental_File
