# Supplementary material for: Printable carbon nanotube superplastics for thermal management
Source: Natl Sci Rev. 2026 Mar 27;13(10):nwag189. doi: 10.1093/nsr/nwag189 (PMC13211993; doi:10.1093/nsr/nwag189)
Supplement: nwag189_Supplemental_File [file nwag189_supplemental_file.pdf]

Supplementary Information

Printable carbon nanotube superplastics for thermal management

Li Chen[1,2,†], Xiangzheng Jia[3,†], Huili Fu[2,†], Zhengpeng Yang[4,†], Yongyi Zhang[1,2,5]*, Dapeng Liu[2], Xinyin Yang[4,7,8], Jiahao Zhan[1,2], Jiangtao Di[1], Liming Zhao[2], Yufang Cao[2], Kunjie Wu[2], Zhenzhong Yong[2,5], Shaoli Fang[6,*], Muqiang Jian[8,*], Enlai Gao[3,*], Jin Zhang[7,8,9,*], Qingwen Li[1,2,*] and Ray H. Baughman[6]

[1]School of Nano-Tech and Nano-Bionics, University of Science and Technology of China, Hefei 230026, China;

[2]Key Laboratory of Multifunctional Nanomaterials and Smart Systems, Suzhou Institute of Nano-Tech and Nano-Bionics, Chinese Academy of Sciences, Suzhou 215123, China;

[3]Department of Engineering Mechanics, School of Civil Engineering, Wuhan University, Wuhan 430072, China;

[4]Henan Key Laboratory of Materials on Deep-Earth Engineering, School of Materials Science and Engineering, Henan Polytechnic University, Jiaozuo 454003, China;

[5]Division of Nanomaterials and Jiangxi Key Lab of Carbonene Materials, Jiangxi Institute of Nanotechnology, Nanchang 330200, China;

[6]AlanG. MacDiarmid NanoTech Institute, University of Texas at Dallas, Richardson, TX 75080, USA;

[7]Beijing Science and Engineering Center for Nanocarbons, Beijing National Laboratory for Molecular Sciences, College of Chemistry and Molecular Engineering, Peking University, Beijing 100871, China;

[8]Beijing Graphene Institute (BGI), Beijing 100095, China;

[9]School of Materials Science and Engineering, Peking University, Beijing 100871, China

***Corresponding authors.** E-mails: yyzhang2011@sinano.ac.cn; sfang@utdallas.edu; jianmq-cnc@pku.edu.cn; enlaigao@whu.edu.cn; jinzhang@pku.edu.cn; qwli2007@sinano.ac.cn

[†]Equally contributed to this work.

1. Experimental

1.1 Materials

PA6, PAN, PVP, PEKK, and PC were purchased from *Badische Anilin-und-Soda-Fabrik* (BASF) China (Mainland); Shanghai Aladdin Biochemical Technology Co., Ltd.; Shandong Xiya Chemical Industry Co., Ltd.; Shandong Kaisheng New Materials Co., Ltd.; and Jilin Zhongyan Polymer Materials Co., Ltd., respectively. Formic acid (HCOOH) (≥ 99%), acetone (CH_3COCH_3) (≥ 99%), and ethanol (C_2H_5OH) (≥ 98%) were purchased from Sinopharm Chemical Reagents Co., Ltd. Thiophene (C_4H_4S) (≥ 99%) and ferrocene ($Fe(C_5H_5)_2$) (≥ 99%) were purchased from TCI (Shanghai) Chemical Industry Development Co., Ltd. Dichloroacetic acid ($C_2H_2Cl_2O_2$) (99%) and N,N-dimethylformamide ($HCON(CH_3)_2$) (> 99%) were obtained from Shanghai Macklin Biochemical Technology Co., Ltd. All reagents were used without further purification. Deionized water (DIW) (resistivity > 18 MΩ cm^{-1}) was collected by a water purification system from Fuchengxin (Wuxi) Technology Co., Ltd.

1.2 Fabrication of CNTSPs

The PA6-CNTSPs were prepared by the FCCVD. An acetone reaction solution containing 0.5 wt% ferrocene and 1.0 wt% thiophene was injected uniformly at the top of a 100-mm-diameter vertical ceramic reactor tube, where this reaction solution composition was optimized to obtain long CNTs. A mixture of hydrogen and argon with a volume ratio of 1:1 was used as the carrier gas and the total gas flow was set at 5 L min^{-1}. The injection rate of the reaction solution and the growth temperature were set at 30 mL per hour and 1300°C. The long CNTs were formed in the high-temperature reaction zone through catalytic growth and further integrated into a sock-like hollow aerogel. Then, the CNT network was continuously immersed into different PA6/formic acid solutions having PA6 concentrations ranging from 0.2 wt%-8 wt% (with a collection speed of 5 m min^{-1}), and then the resultant fiber was continuously dried at 280°C in a tube furnace (with a collection speed of 1.5 m min^{-1}). The thereby produced PA6-composited CNTs were then subjected to multilevel hot drawing (210°C) by drawing through a mechanical die and then converted into a ribbon by hot rolling (210°C) with a collection speed of 1.5 m min^{-1}. By regulating the concentration of PA6 in the solution, we obtained CNTSPs with different CNT mass fractions

(17, 20, 23, 25, 28, 33, 35, 48, 59, and 74 wt%). The fabricated CNTSPs were then processed into various shapes by hot drawing using the corresponding drawing dies.

To prepare the PEKK-CNTSPs, PVP-CNTSPs, PC-CNTSPs, and PAN-CNTSPs, the sock-like hollow aerogel (prepared by the above-mentioned method) was continuously immersed into PEKK/dichloroacetic acid, PVP/ethanol, PC/N,N-dimethylformamide, and PAN/N,N-dimethylformamide solutions, respectively. The PEKK, PVP, PC, and PAN mass fractions were either 2 wt% or 5 wt%. Afterwards, the wet-state polymer/CNT network was dried at 280℃ in a tube furnace. The resulting dry-state polymer/CNT network was then subjected to multilevel hot drawing at 340℃, 130℃, 220℃, and 320℃ for PEKK/CNT, PVP/CNT, PC/CNT, and PAN/CNT networks, respectively. Afterwards, the networks were converted into ribbons by hot rolling at 320℃, 120℃, 210℃, and 300℃ for PEKK/CNT, PVP/CNT, PC/CNT, and PAN/CNT networks, respectively. The CNT mass fractions of these CNTSPs were characterized by thermogravimetric analysis (TGA). The overall fiber collection speeds for conducting these consecutive processes were the same as the 1.5 m min^{-1} or 5 m min^{-1} used in the fabrication of PA6-CNTSPs.

1.3 Fabrication of CNTSP fibers and films

Fibrous CNTSPs were produced using the aforementioned preparation method, involving multilevel hot drawing with drawing dies, yielding fibers extending to lengths of hundreds of meters. CNTSP films with different CNT mass fractions from 0 wt% to 74 wt% were fabricated by aligning multiple CNTSP ribbons side by side in the lateral direction and hot-rolling at 210℃.

1.4 Fabrication of CNT ribbons and films

The CNT ribbon was prepared by the FCCVD. Following the above-mentioned processes, we also fabricated CNT ribbons by changing the solution to a formic acid solution, followed by drying at 280℃ in a tube furnace (with a collection speed of 1.5 m min^{-1}). Based on above-mentioned processes, we used a spool with a diameter of 3 cm and a length of 8 cm. The wet-state CNT assembly was collected on this reel at a lateral speed of 0.2 m min^{-1} and a winding speed of 5 m min^{-1}. Subsequently, the CNT film was obtained by drying in a 100℃ air-circulating oven for 1 hour.

1.5 Fabrication of PA6 ribbons and films

A 200 g PA6 masterbatch was dried in an 80°C air-circulating oven for 24 hours. The dried PA6 was then fed into a twin-screw extruder (DLX_TCSA6, Hangzhou Delixi Group Co., Ltd) using an extrusion speed of 5 m min^{-1}. Following hot rolling at 245°C with a collection speed of 1.5 m min^{-1}, PA6 ribbons were produced for comparison with CNTSP ribbons in mechanical properties. PA6 films were fabricated by aligning multiple PA6 ribbons side by side and hot-rolling at 210°C with a roller gap of 23 μm.

2. Characterization

Wide-angle x-ray scattering measurements: WAXS images were obtained on a two-dimensional (2D) WAXS system using Cu-Kα radiation (Xeuss 3.0, Xenocs). The scattering vector is $q = 4\pi\sin\theta/\lambda$, where θ is the scattering angle and λ is the wavelength of an incident x-ray beam (1.54189 Å). The distance between the sample and the detector was 50 mm. The alignment order parameter (Herman's factor, *f*) [1] is:

$$f = \frac{3\cos^2\varphi - 1}{2}, \quad (1)$$

where φ is the angle between the fiber axis and the crystal plane, and $\cos^2\varphi$ is the average value of the square of the cosine of the azimuthal angle of CNTSPs, which is calculated as follows:

$$\cos^2\varphi = \frac{\int_0^{\pi/2} I(\varphi)\cos^2\varphi\sin\varphi\,\mathrm{d}\varphi}{\int_0^{\pi/2} I(\varphi)\sin\varphi\,\mathrm{d}\varphi}, \quad (2)$$

where $I(\varphi)$ is the intensity at an azimuthal angle of φ.

The analysis of WAXS patterns was performed using FIT 2D software. WAXS was used to measure the orientation of CNT in CNTSP ribbons.

Stress relaxation measurements: Stress relaxation measurements were conducted by stretching CNTSP and PA6 ribbons to 1.5% strain at a loading rate of 1 mm min^{-1} using an Instron 3365 mechanical testing machine with a 100 N load cell. This stretching was in the CNT orientation direction for CNTSP ribbons. The dependence of stress on time was recorded while the strain was maintained.

Mechanical properties: Tensile stress-strain curves of different CNTSP ribbons were obtained using a loading rate of 1 mm min^{-1} (Instron 3365, USA with a 100 N load cell) at a temperature of

27°C under 18% humidity. The thickness and width of CNTSP ribbons were determined by taking average measurements on sample strips at 10 different positions. The indicated uncertainties in measurement results are plus or minus one standard deviation. The samples were attached using cyanoacrylate glue (Zhejiang Guosen Fine Chemical Technology Co., Ltd) to a paper frame with a rectangular hole (10 mm × 10 mm), and the size of the hole was sufficient to accommodate the gauge length and sample width. This rigid frame served as a protective barrier to prevent damage to the sample during attachment to the testing apparatus. After attaching the sample, the two legs of the frame were cut to release the sample for tensile measurements. The modulus was equal to the slope of curves at 0.5%-1% strain. The average mechanical properties (tensile strength and Young's modulus) and their corresponding standard deviations were calculated based on 7-10 measurement results. The statistics did not include measurement results for samples that fractured near the edge of the paper frame.

Electrical properties: A digit multimeter (DMM7510) was used to measure the electrical resistivity. At a temperature of 27°C under 18% humidity, the samples were attached to a paper frame with a rectangular hole (1 cm × 10 cm). The length and width of the hole were sufficient to accommodate the gauge length and sample width. Conductive silver paste (Guangzhou Kaixiang Electronic Products Co., Ltd) was used to ensure low resistivity between the measuring electrical equipment and the sample-contacting silver paste. The average electrical resistivity and its corresponding standard deviation were calculated based on 7-10 measurements. Electrical conductivity (σ) was defined as the following:

$$\sigma = \frac{1}{\rho}, \tag{3}$$

$$\rho = \frac{RS}{L}, \tag{4}$$

where ρ is the electrical resistivity, R, S, and L refer to resistance, cross-sectional area, and length of CNTSP ribbons, respectively.

Thermal conductivity of the CNTSP ribbon in the CNT orientation direction: κ_{max} was measured using a self-heating 3ω method [2]. A schematic diagram of the self-heating 3ω method is shown in Fig. S20a, which has been verified as an appropriate tool to extract κ_{max}. The signal generator provides an AC sine wave with a frequency of ω, which serves as the reference signal

for the lock-in amplifier and the excitation signal for the test sample. Due to the Joule effect, the sample generates a 2ω thermal wave signal. The 3ω signal obtained by superimposing the excitation ω signal and the 2ω thermal wave signal contains the thermal properties of the sample. This 3ω signal was detected by the sample and sent to the lock-in amplifier. After differentiation, the effective signal at the same frequency as the reference signal was extracted by the lock-in amplifier. After subsequent data processing, the thermal conductivity of the sample was determined using the following equations:

$$\kappa_{\max} = \frac{4U_{1\omega}^{3}\alpha_{\mathrm{R}} L}{\pi^{4} RSU_{3\omega}\sqrt{1+\left(\tan\theta\right)^{2}}} \text{ and } \tag{5}$$

$$\alpha_{\mathrm{R}} = \frac{R'}{R}, \tag{6}$$

where U is the harmonic voltage, α_{R} is the temperature coefficient of resistance, and L, R, and S represent sample length, resistance, and cross-sectional area, respectively. The results ($\kappa_{\max}$) are the intrinsic thermal conductivity of CNTSP ribbons in the highly conducting direction.

Before the experiment, a single platinum wire with a purity of 99.99% and a diameter of 15 μm was placed on four raised pads using gold wire bonding technology (Fig. S20b) to ensure that the substrate had no effect on the measurement results. We used harmonic detection technology to obtain the fundamental and third harmonic signals of the platinum wire between the two middle pads. The series model of the third harmonic voltage in the length direction of the heating wire during AC heating in the frequency domain (Eq. 5) was used to calculate the thermal conductivity $\kappa_{\max}$ from the effective value and phase angle of the third harmonic at a low frequency [3]. The measurement error of the system was less than 5%. Fig. S20c shows the experimental system and the sample structure of the 3ω method used for this measurement. The thickness and width were determined by taking average measurements of sample strips at 10 different positions using a high-power microscope.

Through-plane thermal conductivity of the CNTSP ribbon: $\kappa_{\min}$ was measured using a light flash system (LFA 467 NanoFlash). A schematic diagram of the light flash system is shown in Fig. S22a. The LFA 467 HyperFlash is designed as a vertical system with the light source at the bottom, the sample in the center, and the detector on top. A xenon lamp served as the flash source. During the process, the constant temperature and insulation conditions of the material were controlled by

the furnace. Heating elements or liquid nitrogen were used to measure the thermal diffusion coefficient at different temperatures, and a vacuum pump was utilized to provide a vacuum in the furnace, so that an ideal insulation condition was obtained. A light source emitted light pulses to heat the sample, while infrared detectors were employed to monitor temperature variations. The laser pulse is irradiated from the bottom onto the lower surface of the sample located in the middle position, causing the lower surface of the sample to instantly absorb energy and be heated. The samples in this experiment were prepared by cutting CNTSP film into a 2.5 cm-diameter circular shape using a mold (Fig. S22b and 22c).

Mathematical analysis of the measured temperature/time function allows the determination of the thermal diffusivity *a*. For the used adiabatic surface heating condition, *a* is determined by the Parker-formula:

$$a = 0.1388 \frac{s^2}{t_{1/2}}, \tag{7}$$

where s is the thickness of the film sample and $t_{1/2}$ is one-half of the time required for the top surface to reach a constant temperature. The sample's specific heat c_p was measured by differential scanning calorimetry. Using this result for c_p, the measured thermal diffusivity (a), and the measured film density (ρ), the film's thermal conductivity perpendicular to the CNT orientation direction ($\kappa_{\min}$) is given by:

$$\kappa_{\min} = a\rho c_p . \tag{8}$$

Other characterizations: To enable cross-sectional observation of CNTSPs, a focused ion beam was employed using a Hitachi IM4000II. The acceleration voltage was 5 kV and the current decreased from 380 μA to 180 μA. SEM images were obtained using a field emission scanning electron microanalyzer (Apreo C HiVac) with an acceleration voltage of 10 kV. EDS mapping using K line x-ray was conducted using the Burker EDS system with an Xflash 6/60 detector. TEM images were taken using a Talos F200X instrument at 200 kV. For TEM observations, the samples were first cut in the thickness direction using a gallium ion beam and the Thermofisher Helios 5UX SEM was used. This process enabled precise removal of materials, resulting in a 60 nm-thick test sample. Then, the test samples were soldered onto copper grids. The contact angles (CA) between droplets (PA6/formic acid, PEKK/dichloroacetic acid, PVP/ethanol, PC/N,N-dimethylformamide, and PAN/N,N-dimethylformamide solutions) and the CNT film were measured by OCA 15EC DataPhysics Instruments GmbH. Infrared photos were taken with a Fluke

(TI480 Pro) camera. Polarized Raman spectroscopy (Horiba LabRAM HR Evolution) was used to analyze the graphitization degree and orientation of the CNTSPs.

Nanoscale x-ray computed tomography (nano-CT) was performed using a SkyScan 2211 with a Cu-Kα x-ray source. Tomographic scanning was executed at a resolution of 200 nm, with 971 projections gathered over a 180° rotation (at intervals of 0.2°), employing 2 × 2 binning and an exposure time of 950 ms. The tomographic images were reconstructed using NRecon software (Bruker Corporation). Three-dimensional volume rendering, image segmentation, and statistical analysis were performed by using a Bruker Corporation CTan.

TGA measurements were recorded with an instrument (NETZSCH, TG-209 F1.Germany) under a nitrogen atmosphere from room temperature to 900°C by using a temperature scan rate of 10°C min^{-1}. The mass fraction of CNT in CNTSPs (w) was calculated as the following:

$$w = \frac{M_{\mathrm{CNTSPs}} - M_{\mathrm{PA6}}}{M_{\mathrm{CNT}} - M_{\mathrm{PA6}}}, \tag{9}$$

where M_{CNTSPs}, M_{CNT}, and M_{PA6} are the weight loss calculated from TGA curves of CNTSPs, CNT, and PA6, respectively. The TGA curves and corresponding calculated results are presented in fig. S10. The volume fraction of the CNTs in CNTSPs can be determined using the following equation:

$$\gamma_{\mathrm{CNT}} = \frac{w_{\mathrm{CNT}}\rho_{\mathrm{PA6}}}{w_{\mathrm{CNT}}\rho_{\mathrm{PA6}} + \left(1\text{-}w_{\mathrm{CNT}}\right)\rho_{\mathrm{CNT}}}, \tag{10}$$

where γ is the volume fraction, w is the mass fraction, and ρ refers to the density. The density of CNTs was estimated based on the number of walls and the nanotube diameter [4].

3. Density functional theory-based tight-binding calculations

To evaluate the enhanced binding energy between the CNTs and PA6 chains, three models (including PA6/CNT, CNT/CNT, and PA6/PA6) were studied by placing one of the structures on top of itself. DFTB calculations were then performed using the DFTB+ package [5]. All DFTB computations were performed with DFT-D3 dispersion correction with Becke-Johnson damping [6,7]. The 3-ob-1 Slater-Koster set of parameters was employed [8–10]. In-plane periodic boundary conditions were used, and the vacuum layer with a thickness over 4 nm was adopted along the out-of-plane direction. Taking PA6/CNT as an example, the binding energy was calculated by the sum of the energies of the individual PA6 chain and the isolated CNT (sp^2 carbon

sheet) minus the total energy of the composite system. Then the binding energy was divided by the total number of atoms (except for hydrogen atoms) to obtain the normalized binding energy.

4. Molecular dynamics simulations

The microstructural evolution of materials was explored by molecular dynamics (MD) simulations by using a large-scale atomic/molecular massively parallel simulator (LAMMPS) computational package [11]. The polymer consistent force field (PCFF) was adopted to describe the interatomic potentials [12,13]. The long-range Columbia interaction was included by using the particle-particle-particle mesh (PPPM) method [14], while the van der Waals interaction was described by a 6/9 Lennard-Jones potential. To investigate the Newton equations of motion, the Verlet algorithm was adopted with a time step of 1 fs. For studying the influence of a three-walled CNT on adjacent PA6 chains, 800 PA6 chains were placed randomly around the CNT. Periodic boundary conditions were used along all directions, and the CNT was periodic along the axial direction. The structures were first energy minimized using a conjugate gradient algorithm before the thermostat process. During the thermostat process, the CNT was fixed. The temperature increased from nearly 0 K to 1000 K and then decreased to room temperature of 300 K, which was then equilibrated for 2 ns (Fig. S16).

5. Supplementary Notes

5.1 Affinity between different materials

By quantitatively titrating different solutions (3 μL) on the surface of CNT film prepared by FCCVD, the change in contact angle between droplets and CNT film as a function of time was observed to determine the affinity (Fig. S3). The smaller the contact angle, the stronger the affinity. These results demonstrate that the infiltration between PA6/formic acid, PEKK/dichloroacetic acid, PVP/ethanol, PC/N,N-dimethylformamide, and PAN/N,N-dimethylformamide solutions and CNT is higher than that between water and CNT.

5.2 Effect of hot drawing and hot rolling

To enhance alignment and densification, hot drawing and hot rolling were employed. The dry-state PA6/CNT networks were hot drawn through drawing dies of different sizes until no further

reduction in diameter was observed by SEM. Subsequently, hot rolling was applied to increase packing density. The hot-drawn composites were placed between two rollers heated to 210℃. The rollers were adjusted until no visible gap was observed, and the samples were rolled continuously. The size of the gap can be determined based on the mechanical properties maintained by the CNTSP ribbons after rolling. For instance, when the CNT content was 59 wt%, the rolling gap was 27 μm.

5.3 Effect of CNT mass fraction on processability

We prepared different CNTSPs to explore the effect of CNT mass fraction on processability. First, the SEM image showed that the as-grown CNT network is uniformly immersed into PA6. Then, experimental results showed that as the mass fraction of CNT increased, the mechanical, thermal, and electrical properties of CNTSPs also improved. Since processability is another important characteristic, we quantified the processability of CNTSPs by processing CNTSP fibers of the same length but different CNT mass fractions into helical structures with the same pitch. Then, the stability after processing was determined by analyzing the evolution of pitch over time (Fig. S11, Phenom XL, FEI Electron Optics BV). The results showed that when the CNT mass fraction exceeds 59 wt%, the pitch of the helical structure expands over 10% after 20 h. In comparison, CNTSPs with CNT mass fractions below 59 wt% can maintain their shape with a pitch expansion less than 10%. Therefore, CNTSPs with a mass fraction of CNTs below 59 wt% are the focus of this work.

5.4 Stability of CNTSP ribbon

The structure stability of CNTSPs was examined by measuring the electrical conductivities of CNTSPs after they were exposed to various mechanical and thermal conditions. At a temperature of 27℃ under 18% humidity, the samples were attached to a paper frame with a rectangular hole (1 cm × 5 cm). The length and width of the hole were sufficient to accommodate the gauge length and sample width. Conductive silver paste (Guangzhou Kaixiang Electronic Products Co., Ltd) was used to ensure low resistivity between the measuring electrical equipment (DMM7510) and the sample-contacting silver paste. Afterwards, the two legs of the frame were cut to release the sample for bending. The electrical conductivity of CNTSPs in the CNT orientation direction was little changed during the sharp bending shown in Figure S19A for 10,000 cycles with a bending

angle of 120°. Also, we placed CNTSPs in the thermoelectric refrigeration system (DWB3-50S) to measure the electrical resistivity after standing at the corresponding temperature for 10 minutes. The electrical resistivity in the CNT orientation direction changed by less than 5% when subjected to atmospheric air at temperatures ranging from 25 to 220℃ (Fig. S19B).

5.5 Mechanism of the modified rule-of-mixture

The introduction of the power-law exponent α serves a sensitivity factor to calibrate the reinforcement and transport efficiency of CNT network across different physical domains. In our model, α captures the non-linear synergy between the high-loading CNTs and the PA6 matrix, specifically reflecting the transition from isolated fillers to a continuous, interconnected network. For mechanical properties, α is primarily a reflection of the interfacial load transfer efficiency and the interfacial shear strength. In contrast, for electrical and thermal transport, α represents geometric connectivity and contact resistance within the CNT network. Specifically, in CNTSPs, the non-linear relationship (characterized by α) captures the transition from isolated fillers to a continuous network. As detailed in Table S9, the variation in α across different properties suggests that while the PA6 matrix provides a consistent structural environment, the underlying physics of phonon, electron, and stress propagation respond differently to the CNT volume fraction.

5.6 Formation process of various objects and printouts

Before printing, the 3D structure of the desired part was designed using cinema 4D (C4D) software. The design was saved as an STL file and imported into slicing software to generate a G-code file. This G-code file was then loaded into a self-developed 3D printer for fabricating printouts using CNTSP and Onyx as printing materials. Onyx, which was purchased from Markforged, is a nylon matrix composite reinforced by short carbon fibers, in which the content of short carbon fibers is about 15 vol% and the fiber's diameter is about 8 μm and its length is about 80 μm. During the printing process, the structure of the printed part can be customized by adjusting the number of layers and the stacking sequence of CNTSP and Onyx. To characterize the printing path of CNTSP, the printing process was halted upon completing the CNTSP layer, allowing for optical and SEM analysis (Fig. S25).

6. Supplementary Figures and Tables

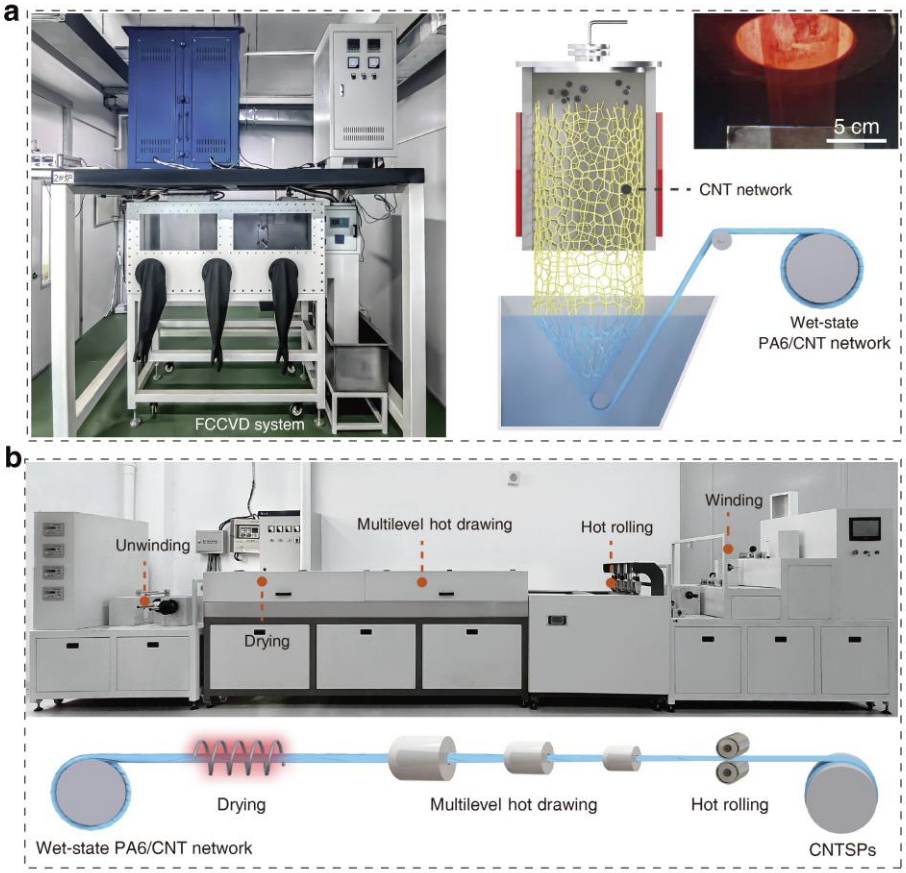

Figure S1. Continuous capture, immersion, and densification processes for preparing CNTSPs. (a) Photograph and schematic diagram of the continuous preparation of CNTSPs using a floating catalyst chemical vapor deposition (FCCVD) system that is in a glove box. The loose CNT networks spontaneously capture and fuse with polymer molecules to form a wet-state polymer/CNT network. The collection speed used was 5 m min^{-1}. (b) Photograph and schematic diagram of the continuous densification process for preparing CNTSP ribbons. The wet-state polymer/CNT network was dried, drawn, and rolled to form a CNTSP ribbon. The collection speed used was 1.5 m min^{-1}.

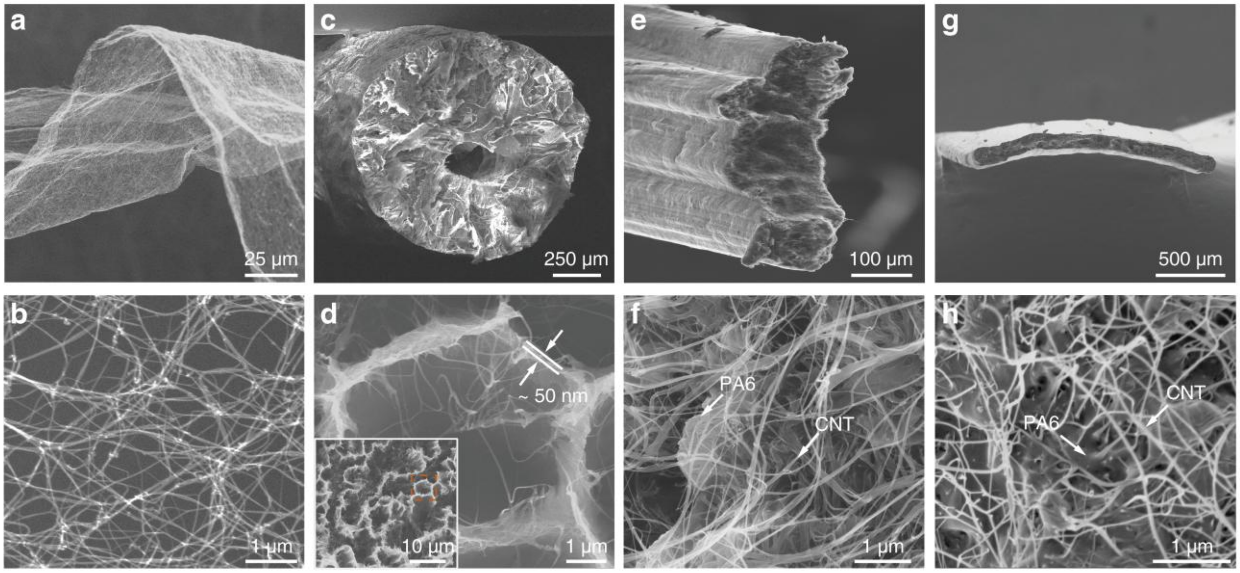

Figure S2. Scanning electron microscope (SEM) images of the structural evolution of CNTSPs during processing. (a) Low-resolution and (b) high-resolution SEM images of loose CNT networks before wet-state spinning. (c) Low-resolution and (d) higher-resolution SEM images of the PA6-composited CNT network after wet-state spinning, where the inset in (d) indicates that the wall thicknesses are about 50 nm. The inset in (d) is a much higher resolution SEM image of the wall indicated by the orange dotted rectangle. (e) Low-resolution and (f) high-resolution SEM images of the wet-spun PA6/CNT network after drying to remove the formic acid, which shows that (f) PA6 molecules in the CNT network are not accessible to each other. (g) Low-resolution and h high-resolution SEM images of the CNTSP ribbon after hot drawing and rolling of the material in (e), which shows that these mechanical processes result in the inter-mixing of PA6 molecules and the CNT network.

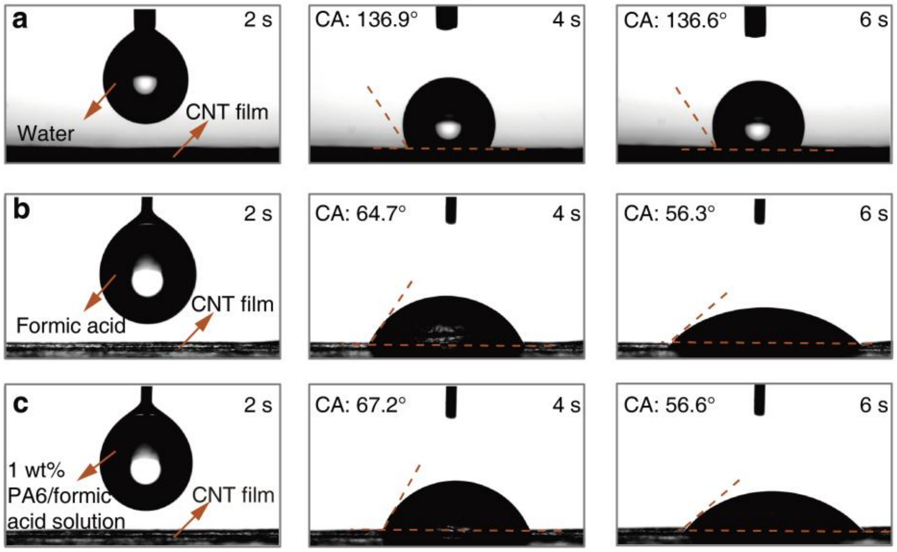

Figure S3. Photographs of the time dependence of contact angle measurements for CNT films. Time-dependent contact angle (CA) measurements of (a) water, (b) formic acid, and (c) 1 wt% PA6/formic acid solution on the surface of a CNT film.

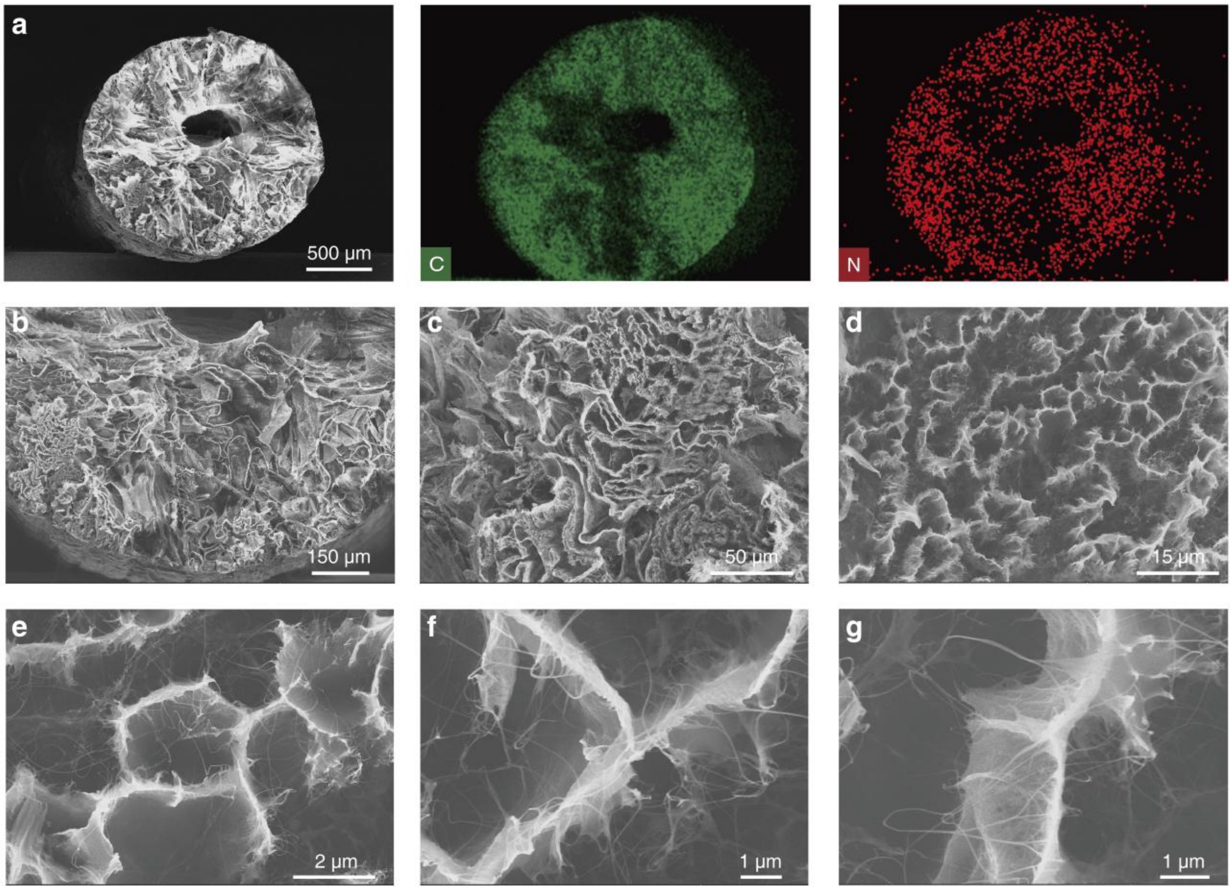

Figure S4. SEM images and element mappings of wet-state PA6/CNT networks before densification. (a) The SEM image of the cross-section (left) and the corresponding energy dispersive spectroscopy (EDS) images (middle and right) for wet-state PA6/CNT networks. Green and red colors in the EDS mapping images represent, respectively, the spatial distributions of C and N atoms. (b-d) Magnified SEM images corresponding to regions in (a). High-resolution SEM images show that the CNT network is preserved by the processing. (e-g) Magnified SEM images corresponding to the enlarged region in (d).

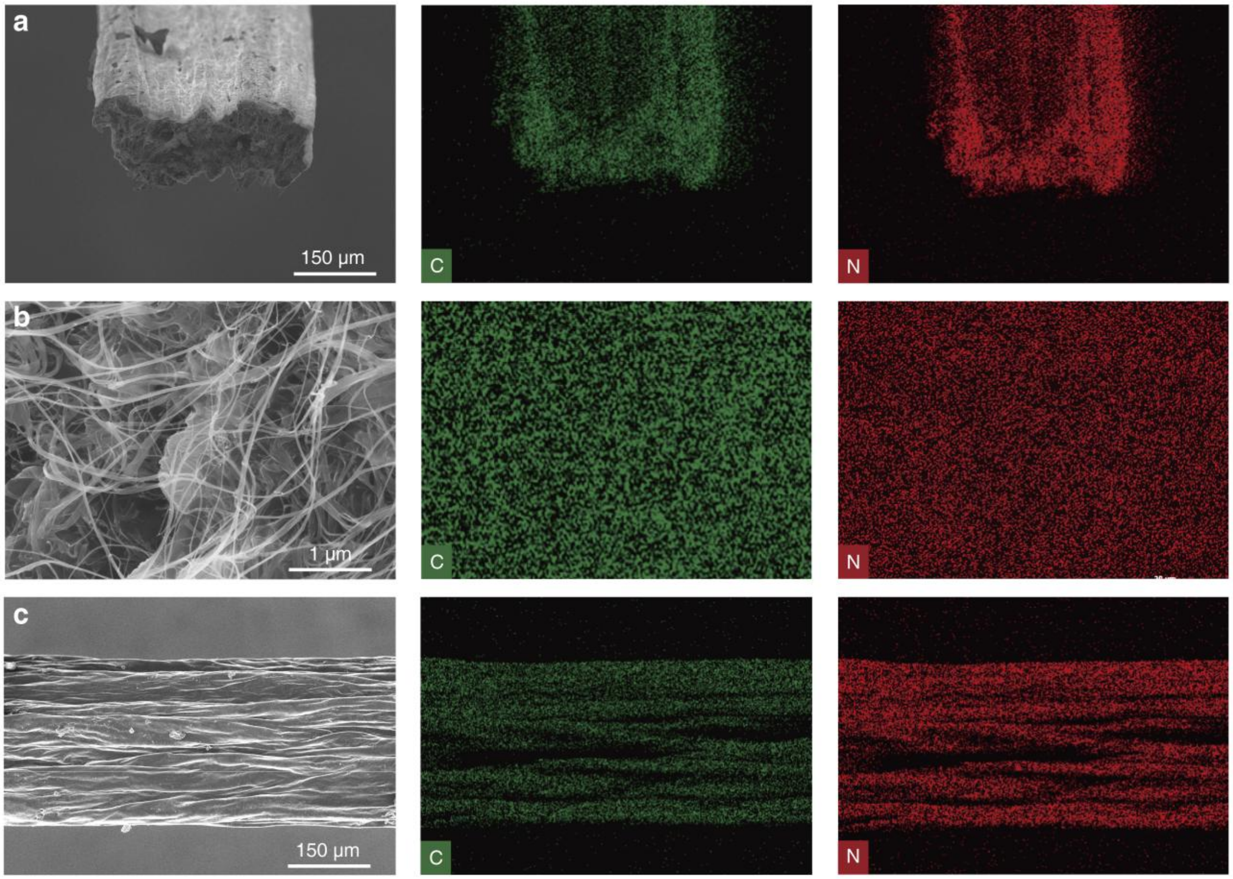

Figure S5. SEM images and element mappings of dry-state PA6/CNT networks. (a) Cross-sectional SEM image (left) and the corresponding elemental mappings of C (green) and N (red) atoms (middle and right, respectively). (b) High-resolution cross-sectional SEM image (left) and the corresponding elemental mappings (middle and right) of the dry-state PA6/CNT networks, showing that PA6 molecules relocated into the CNT network are not accessible to each other after formic acid removal. (c) SEM image (left) of the longitudinal structure and corresponding elemental mappings (middle and right), indicating that PA6 is uniformly dispersed on the surface of dry-state PA6/CNT networks.

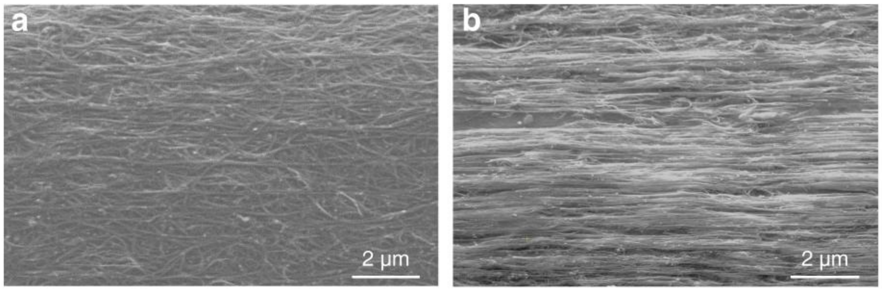

Figure S6. Longitudinal SEM images of (a) a CNTSP array in the dry-state before drawing and (b) after drawing and rolling to produce a CNTSP ribbon.

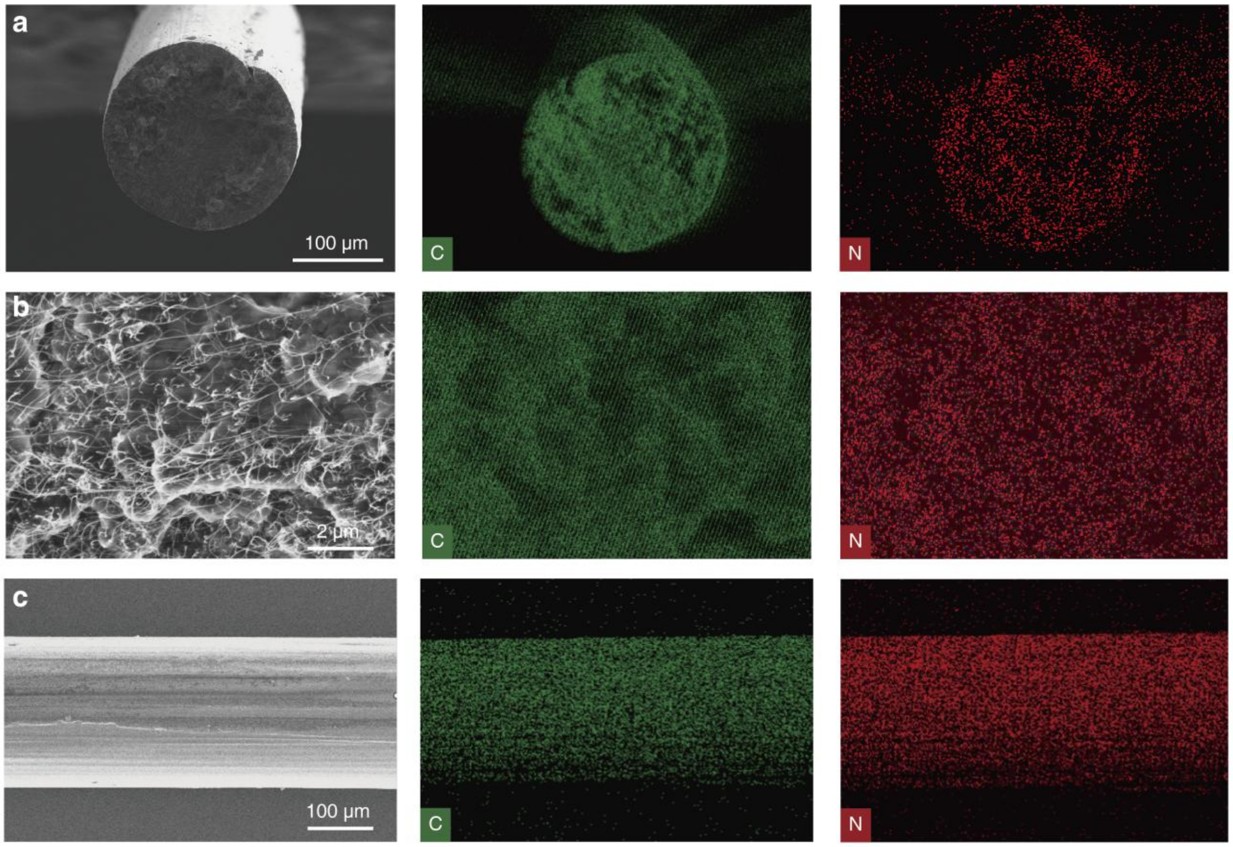

Figure S7. SEM images and elemental mappings of dry-state PA6/CNT networks after multilevel hot drawing, but before the hot rolling in Figure 1a. (a) Cross-sectional SEM image (left) and the corresponding elemental mappings of C (green) and N (red) atoms (middle and right, respectively). (b) High-resolution cross-sectional SEM image (left) and the corresponding elemental mappings (middle and right) of the dry-state PA6/CNT networks after multilevel hot drawing, but before hot rolling. (c) SEM image (left) of the longitudinal structure and corresponding elemental mappings (middle and right), indicating that PA6 is uniformly dispersed on the surface of dry-state PA6/CNT networks after multilevel hot drawing, but before hot rolling.

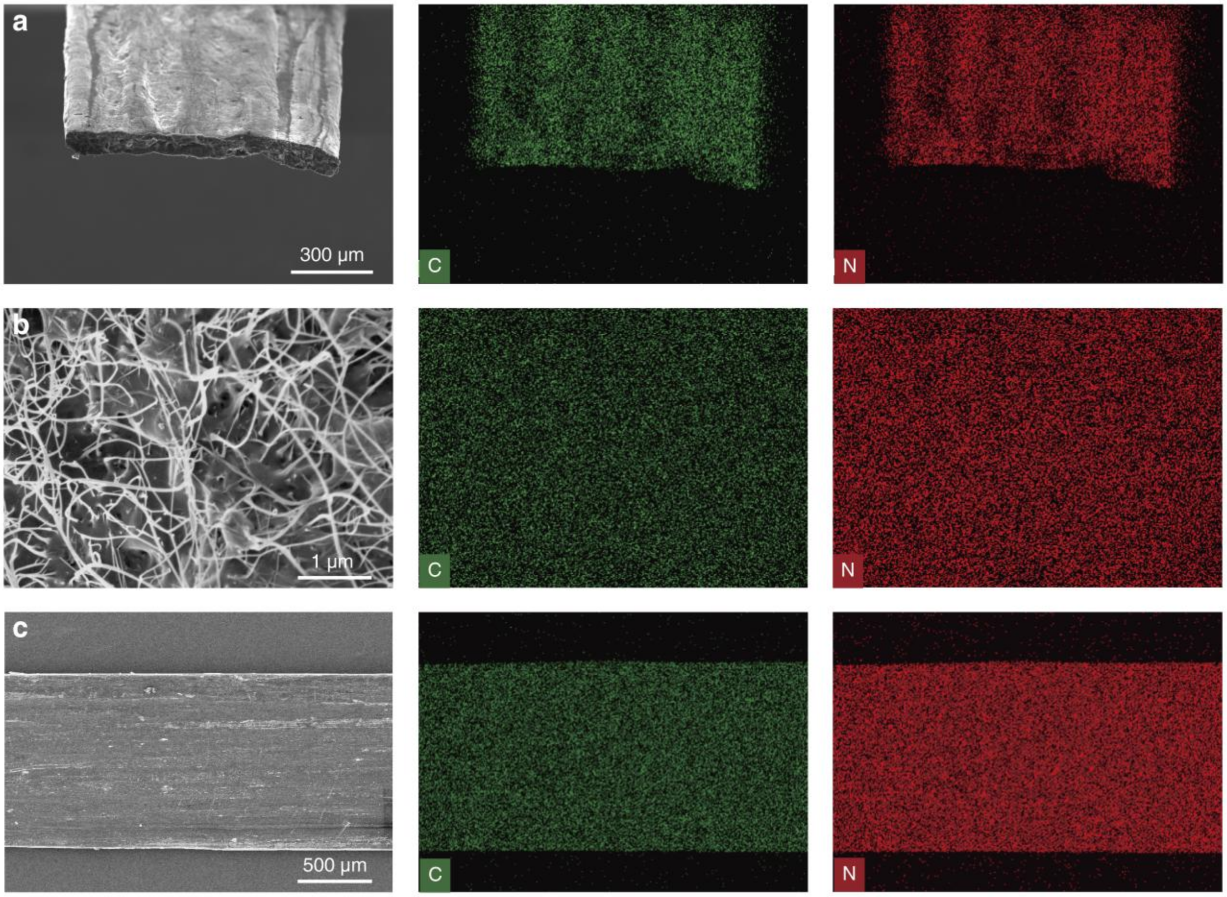

Figure S8. SEM images and elemental mappings of a CNTSP ribbon. (a) Cross-sectional SEM image (left) and the corresponding elemental mappings of C (green) and N (red) atoms (middle and right, respectively). (b) High-resolution cross-sectional SEM image (left) and the corresponding elemental mappings (middle and right) of a CNTSP ribbon, which shows that these mechanical processes result in the inter-mixing of PA6 molecules and the CNT network. (c) SEM image (left) of the longitudinal structure and corresponding elemental mappings (middle and right), indicating that PA6 is uniformly dispersed on the surface of a CNTSP ribbon.

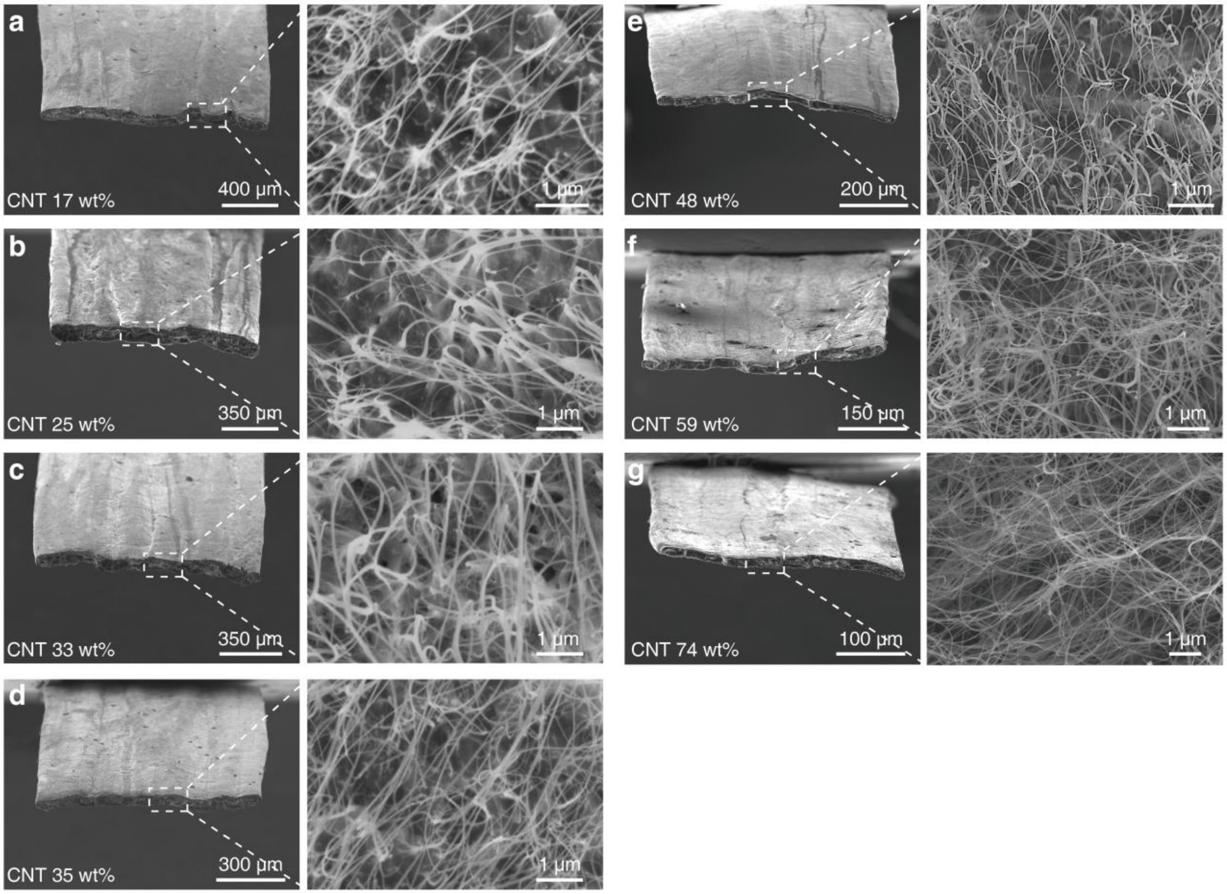

Figure S9. Cross-sectional SEM images of CNTSP ribbons with different CNT mass fractions. Low-resolution (left) and high-resolution (right) cross-sectional SEM images of CNTSP ribbons with CNT mass fractions of (a) 17 wt%, (b) 25 wt%, (c) 33 wt%, (d) 35 wt%, (e) 48 wt%, (f) 59 wt%, and (g) 74 wt%. The low-resolution images are side views of the cross-section and the high-resolution images are orthogonal views of the cross-section.

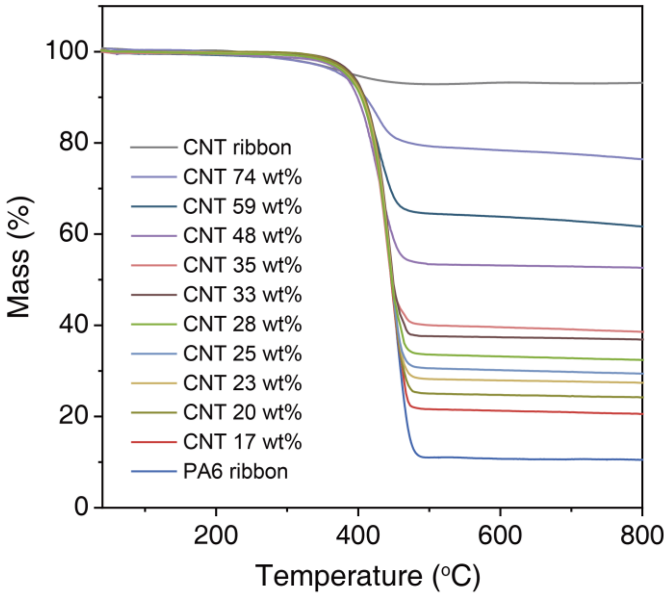

Figure S10. Thermogravimetric analyses (TGA) curves of PA6, CNT, and CNTSP ribbons with different CNT mass fractions. TGA under a nitrogen atmosphere of PA6, CNT, and CNTSP ribbons with CNT mass fractions of 17 wt% to 74 wt%. The temperature scan rate was 10°C minute–1.

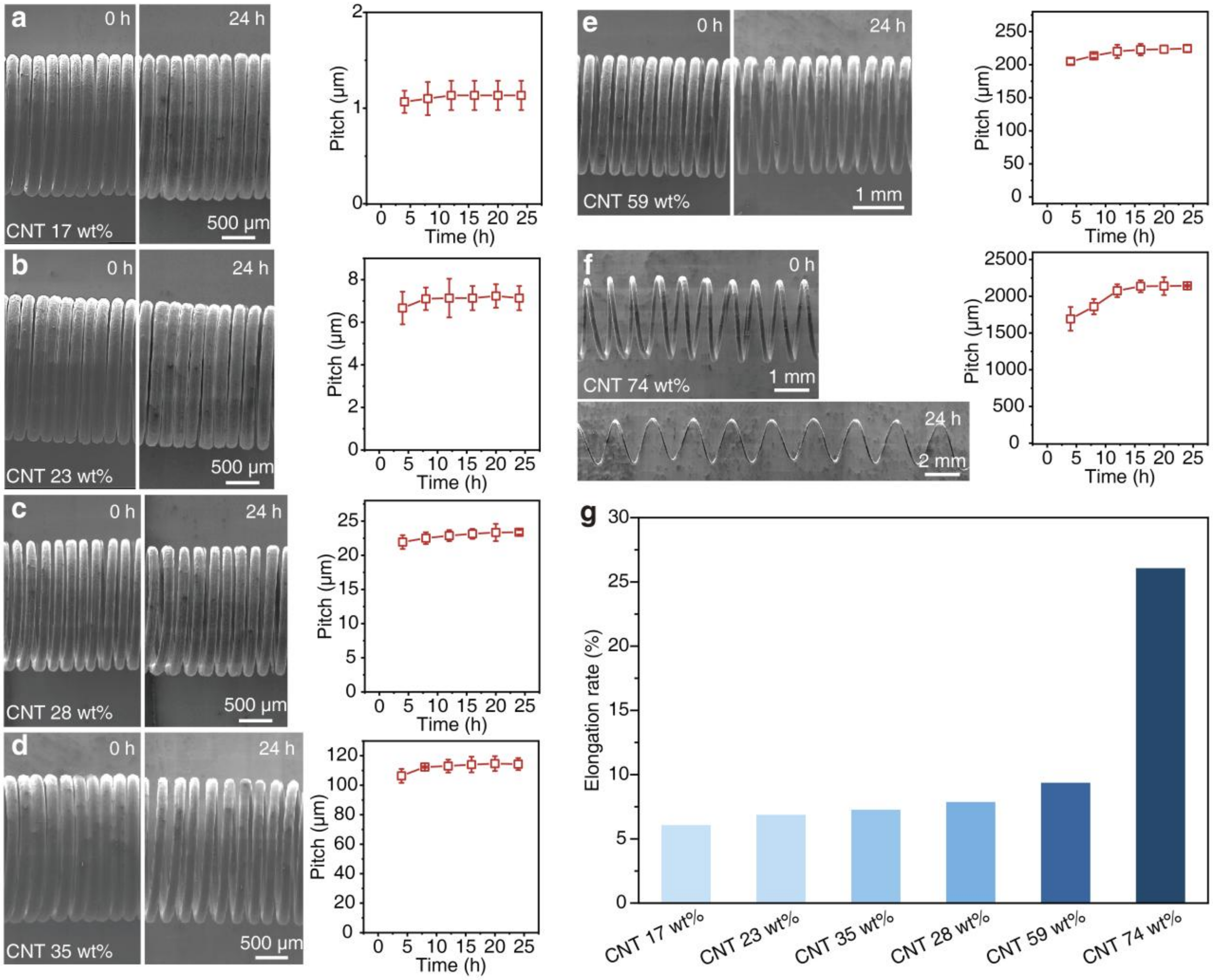

Figure S11. Processability characterization of CNTSP fibers with different CNT mass fractions. SEM images of the initial structures (left) and the structures after 24 hours of relaxation at room temperature (middle), and the corresponding pitch-time curve (right) of CNTSP fibers with CNT mass fractions of (a) 17 wt%, (b) 23 wt%, (c) 28 wt%, (d) 35 wt%, (e) 59 wt%, and (f) 74 wt%. (g) Elongation rate of CNTSP fibers with different CNT mass fractions after 24 hours of relaxation.

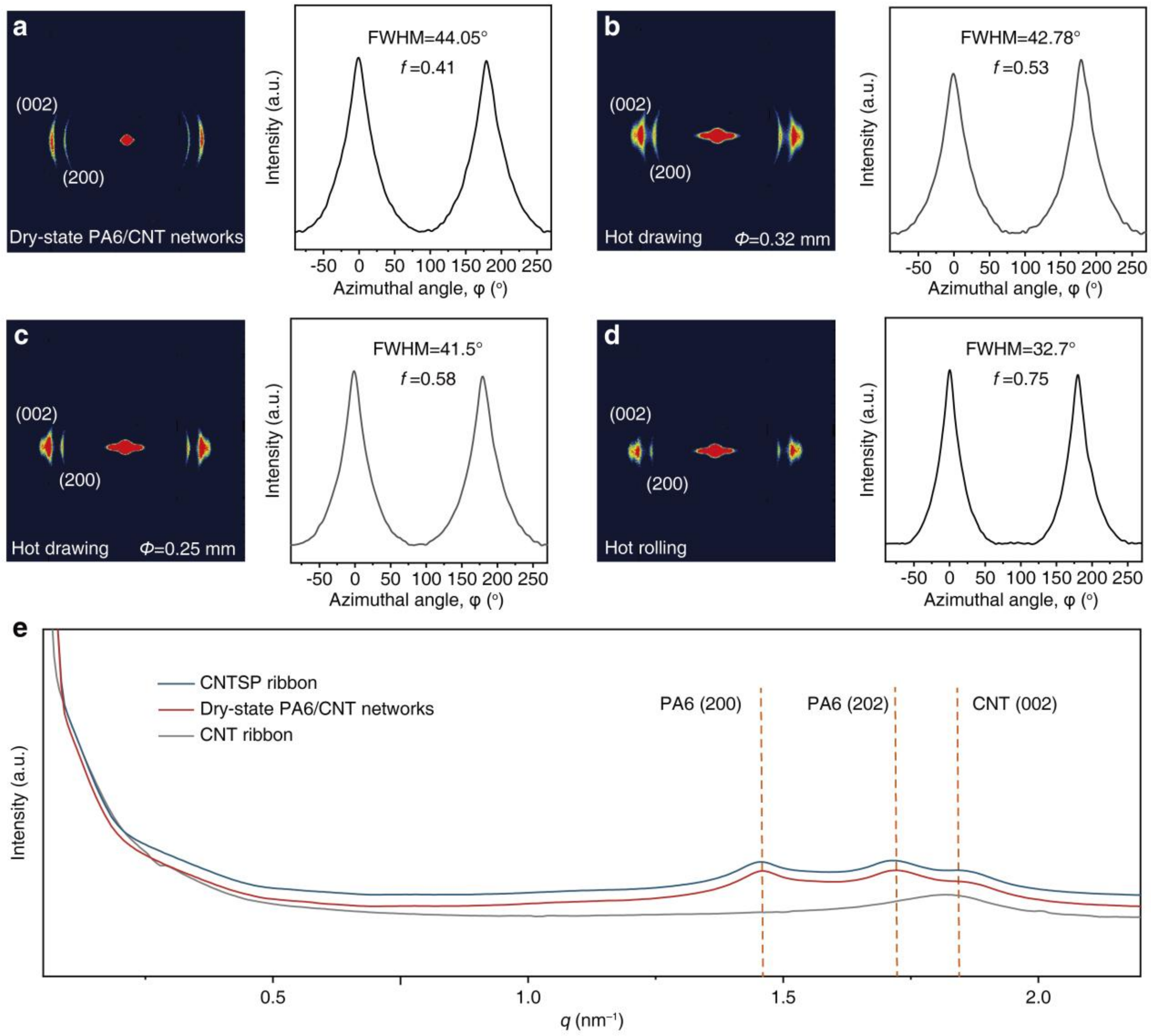

Figure S12. Two-dimensional (2D) wide-angle x-ray scattering (WAXS) analyses of dry-state PA6/CNT networks before and after multilevel hot drawing and rolling. 2D-WAXS patterns (left) and normalized intensity distributions versus azimuthal angle (right) of (a) dry-state PA6/CNT networks, (b-d) networks treated by multilevel hot drawing with different pore sizes and hot rolling. The dry-state PA6/CNT networks were first hot drawn through a 0.32 mm-diameter pore, and then were hot drawn through a 0.25 mm-diameter pore. Finally, the networks were hot rolled with a 27 μm-thick gap. The equatorial radial profile is mainly composed of the (002) plane of CNT and the (200) plane of PA6. As inferred from the equatorial radial profiles, the dry-state PA6/CNT networks have a full width at half maximum (FWHM) of 44.05°. Multilevel hot drawing and rolling are the main transformations that improve the alignment of the CNTs, providing a FWHM of 32.7° and a Herman's factor (f) that can reach 0.75. *Φ* in the figures is the pore diameter of the

dies used for drawing. (e) The scattering intensity of the fiber streak as a function of q along the equator.

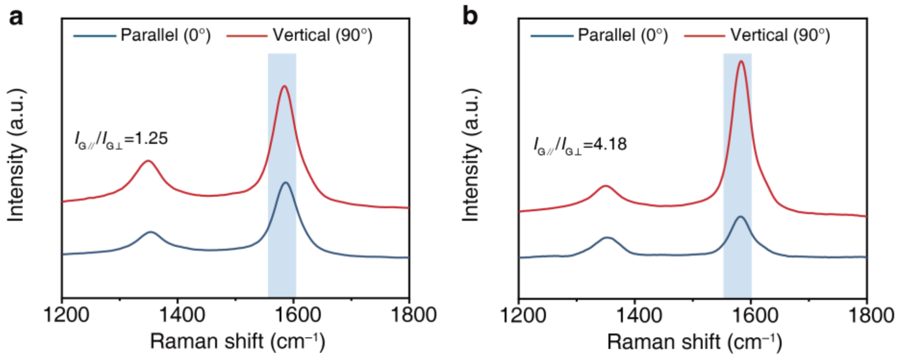

Figure S13. Polarized Raman spectroscopy of CNTSPs before (a) and after (b) hot working and rolling.

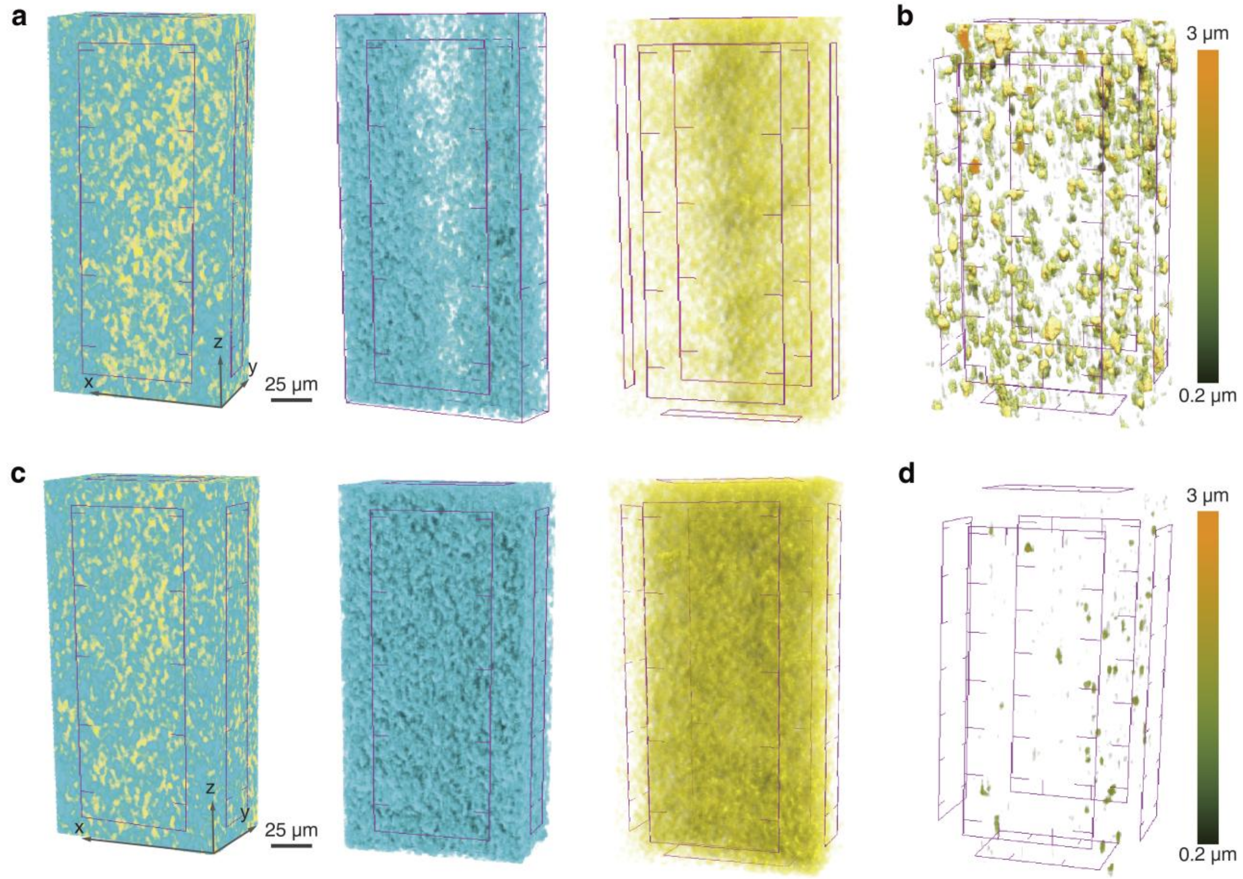

Figure S14. Three-dimensional (3D) reconstruction and void microstructures of dry-state PA6/CNT networks and a CNTSP ribbon by nanoscale x-ray computed tomography (nano-CT). (a) 3D reconstruction and (b) void microstructures of dry-state PA6/CNT networks by nano-CT. (c) 3D reconstruction and (d) void microstructures of a CNTSP ribbon by nano-CT. In (a) and (c), the yellow regions and blue regions represent CNTs and PA6, respectively, while the middle figures and the right figures correspond to 3D PA6 and 3D CNT networks, respectively.

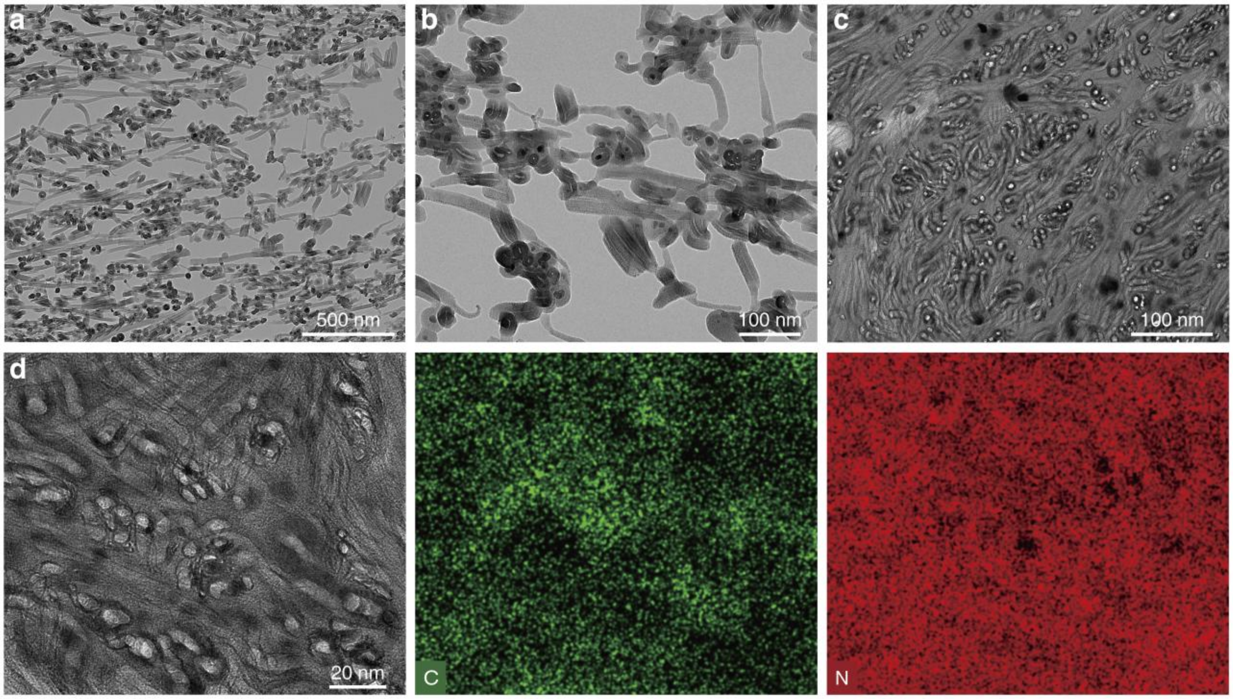

Figure S15. Transmission electron microscope (TEM) images of a CNT ribbon and a CNTSP ribbon. (a) Low-resolution and (b) high-resolution cross-sectional TEM images of a CNT ribbon. (c) Low-resolution and (d) high-resolution cross-sectional TEM images (left) and the corresponding elemental mappings of a CNTSP ribbon (middle and right), showing the spatial distributions of C (green) and N (red) atoms between CNT bundles. These cross-sectional images were derived using a focused ion beam that was perpendicular to the surfaces of the CNTSP ribbon and the CNT ribbon.

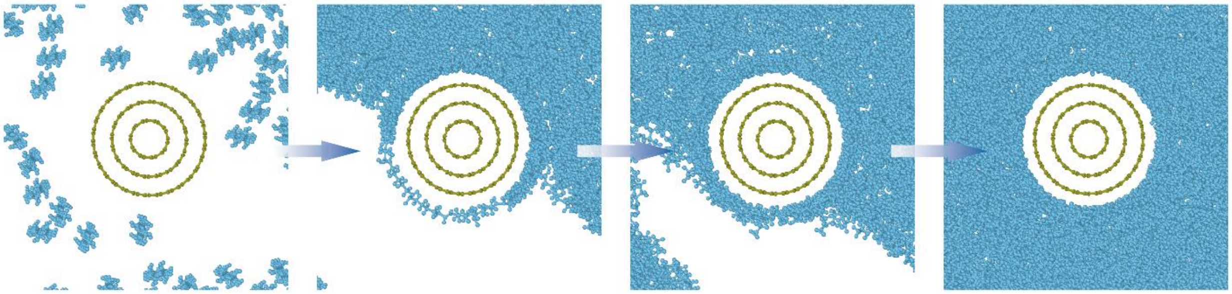

Figure S16. Simulation snapshots for adsorption of PA6 chains on a three-walled CNT surface. The yellow regions and blue regions represent CNT and PA6, respectively.

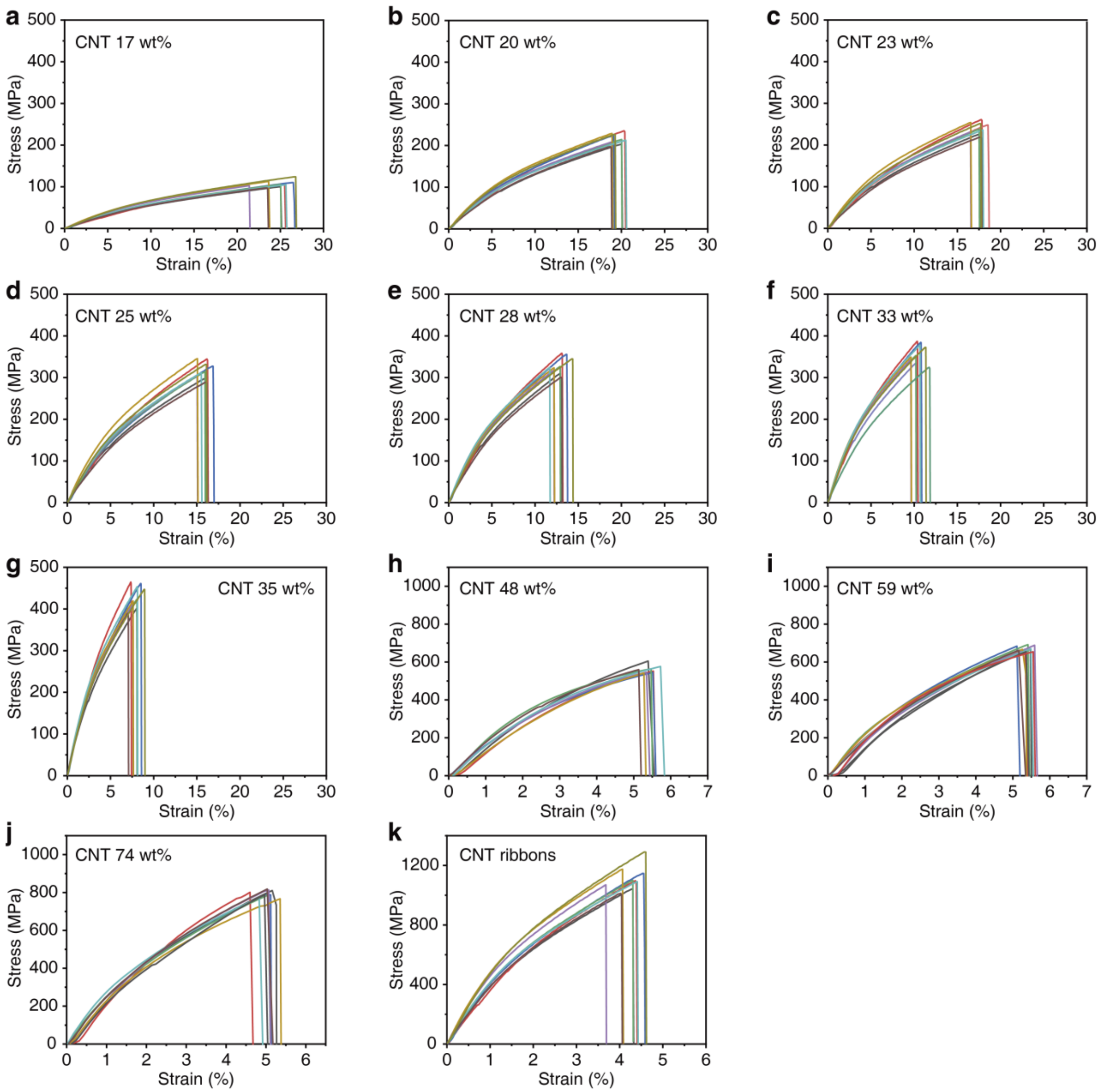

Figure S17. Stress-strain curves based on 7-10 duplicate measurements on CNT ribbons and CNTSP ribbons with different CNT mass fractions. Stress-strain curves in the CNT orientation direction that result from 7-10 duplicate measurements on CNTSP ribbons with mass fractions of (a) 17, (b) 20, (c) 23, (d) 25, (e) 28, (f) 33, (g) 35, (h) 48, (i) 59, and (j) 74 wt%. (k) Stress-strain curves based on 8 duplicate measurements on CNT ribbons.

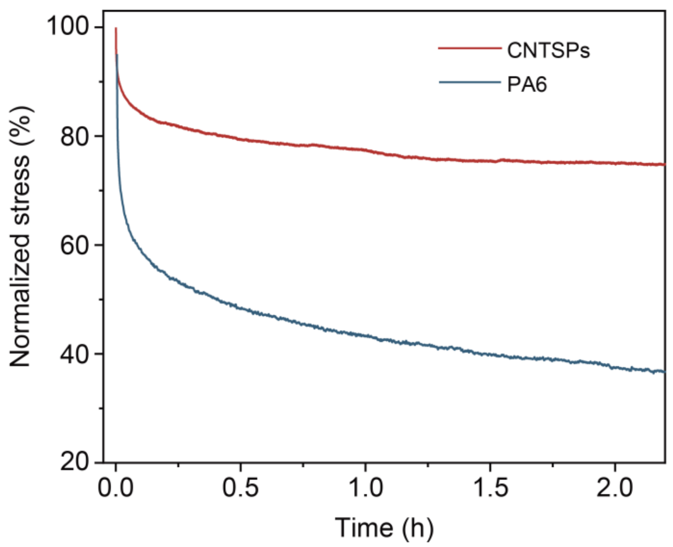

Figure S18. Stress-relaxation curves for PA6 and CNTSP ribbons at a 1.5% strain as a function of the annealing time at room temperature. The stress is normalized to the maximum stress for the non-annealed sample. The CNT mass fraction of the CNTSP ribbon is 59 wt%.

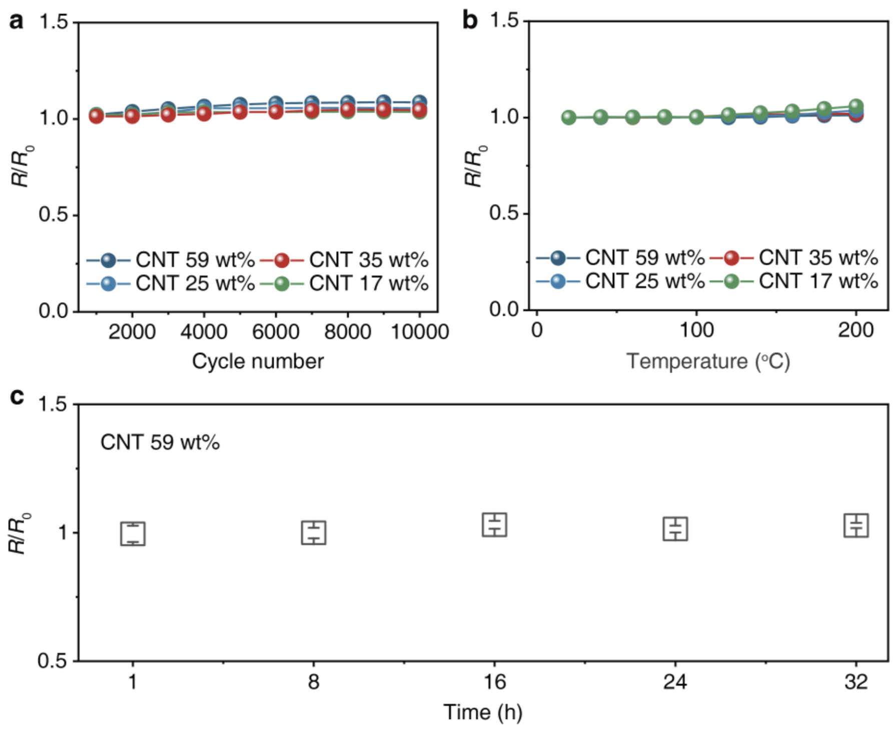

Figure S19. Mechanical and thermal stability of CNTSP ribbons. (a) Relative electrical resistance (R/R_0) of CNTSP ribbons in the CNT orientation direction as a function of bending cycle number for various CNT mass fractions (17, 25, 35, and 59 wt%). R_0 is the initial resistance at room temperature before cycling and R is the resistance after cycling. (b) Temperature dependence of the relative electrical resistance (R/R_0) for CNTSP ribbons (17, 25, 35, and 59 wt%) in the CNT orientation direction. (c) Long-term isothermal stability showing time dependence of R/R_0 in the CNT orientation direction for a 59 wt% CNTSP ribbon at 220 °C over 36 hours, confirming minimal resistance drift under prolonged thermal loading.

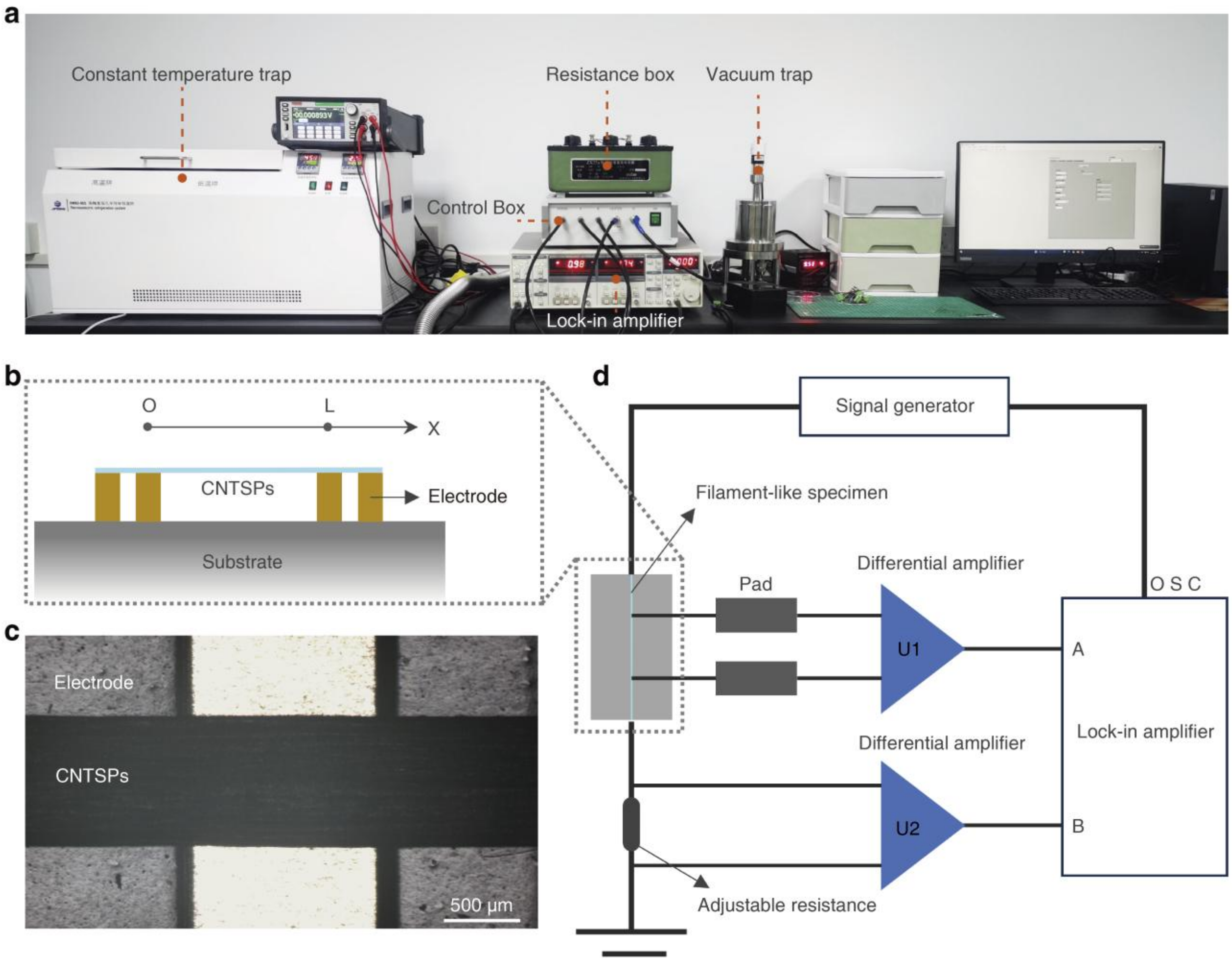

Figure S20. Equipment and schematic diagram of the 3ω method applied for CNTSP ribbons. (a) Photograph of the equipment used for measuring in-plane thermal conductivity by the 3ω method. (b) Illustration of the four-probe configuration for measuring the specific heat and thermal conductivity of CNTSP ribbons. (c) Photograph of a CNTSP ribbon on an electrode platform. d) Schematic diagram of the 3ω thermal property testing system used for CNTSP ribbons.

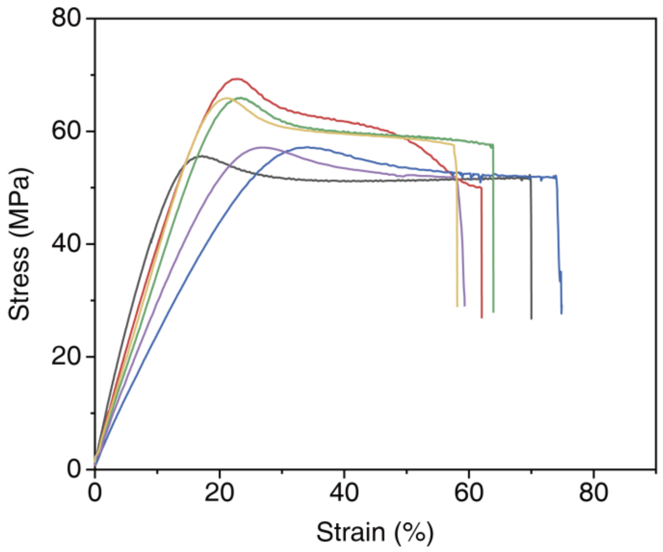

Figure S21. Stress-strain curves based on 6 duplicate measurements on PA6 ribbons.

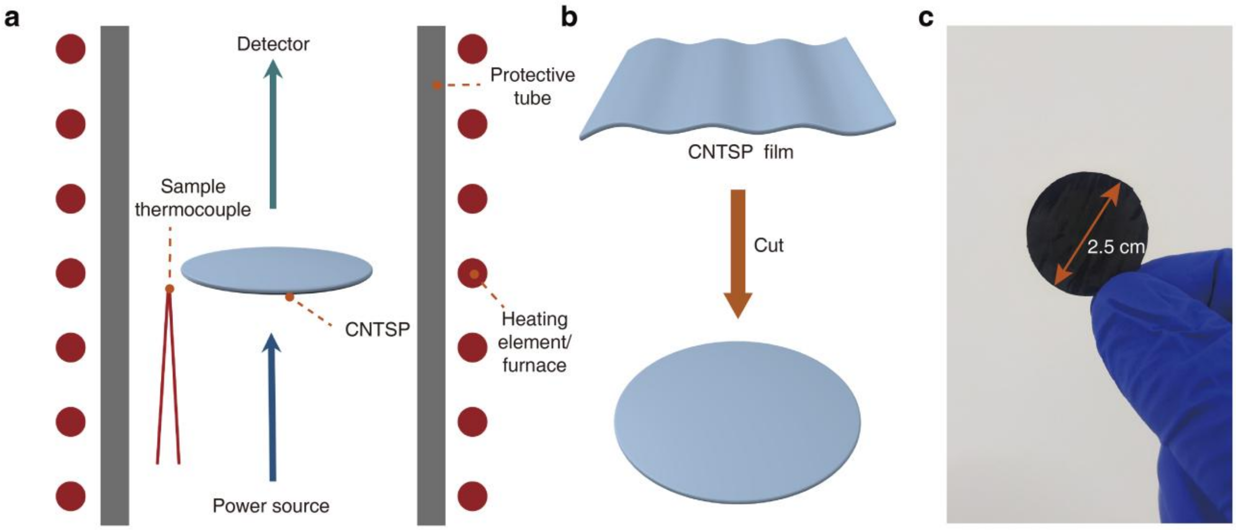

Figure S22. Schematic diagram of the equipment used for measuring thermal conductivity perpendicular to the CNT orientation direction by using a light flash system. (a) Schematic diagram of the light flash system used for thermal conductivity measurements and (b) the fabrication process used for sample preparation. (c) Photograph of a fabricated 2.5-cm-diameter CNTSP film that was 27-μm thick.

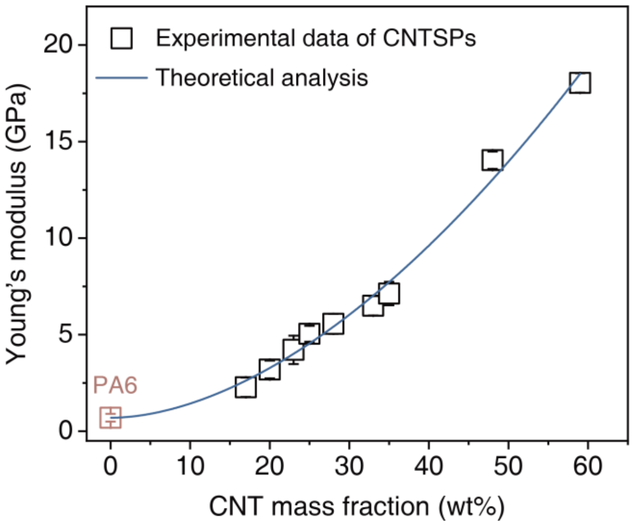

Figure S23. Experimental and theoretical analysis results for the dependence of Young's modulus of CNTSP ribbons on CNT mass fraction. When the CNT mass fraction is 0 wt%, the Young's modulus of PA6 is about 0.7 GPa. With the increase of CNT mass fraction, Young's modulus of CNTSPs in the CNT orientation direction increases, which is consistent with theoretical analysis based on our rule-of-mixtures.

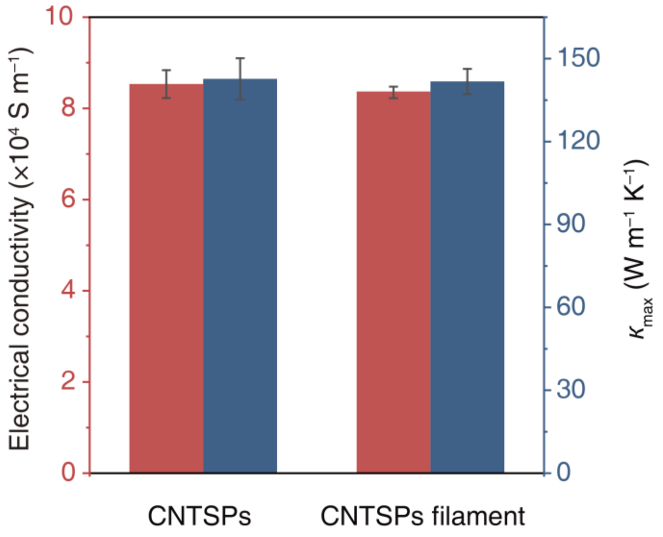

Figure S24. Property retention during the hot-drawing process. Comparison of thermal and electrical conductivities measured along the printing direction for a CNTSP ribbon and a single 400 μm diameter CNTSP filament.

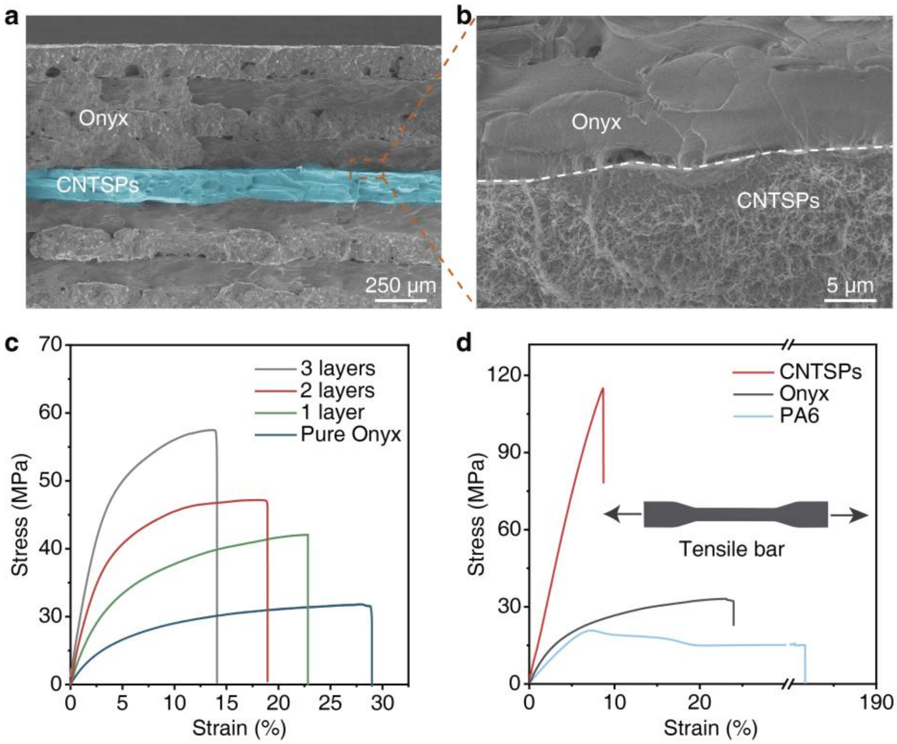

Figure S25. Cross-sectional structure characterization and mechanical properties of tensile bars printed on Onyx, PA6, and CNTSP substrates. (a) Low-resolution and (b) high-resolution cross-sectional SEM images of a one-layer CNTSP printout of CNTSP on an Onyx substrate. (c) Typical tensile stress-strain curves of Onyx and CNTSP printouts with different numbers of printed CNTSP layers, which alternate with an Onyx layer. (d) Typical stress-strain curves of CNTSP, Onyx, and PA6 tensile bars using a tensile strain rate of 3 mm min–1, where the interlayer separating substrate used for printing was either Onyx or PA6. In other words, the initial substrate printed was either Onyx or PA6, then after printing either a layer of CNTSP, Onyx, or PA6, another layer of CNTSP, Onyx, or PA6 was printed, and then this process was repeated until a total of 10 layers of printed material was obtained.

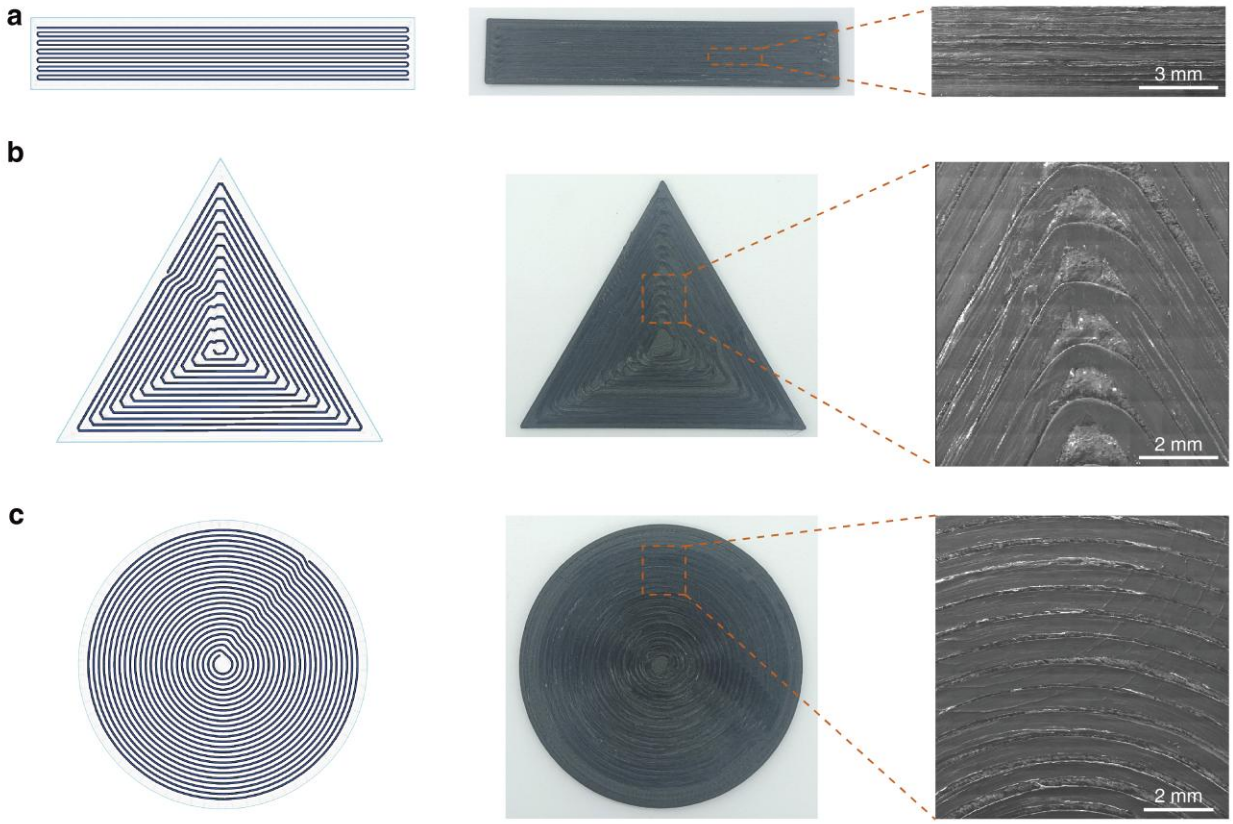

Figure S26. Images of the computer inputted structure (left), photographs (middle), and SEM images (right) of CNTSP printouts for printing continuous adjacent paths of an Onyx layer and a CNTSP layer to obtain either (a) straight, (b) triangular, or (c) round-shaped printouts. The magnified SEM images showed that the 3D printouts were continuous at the corners, indicating that the CNTSP filament laying process did not break the integrity of the CNT network.

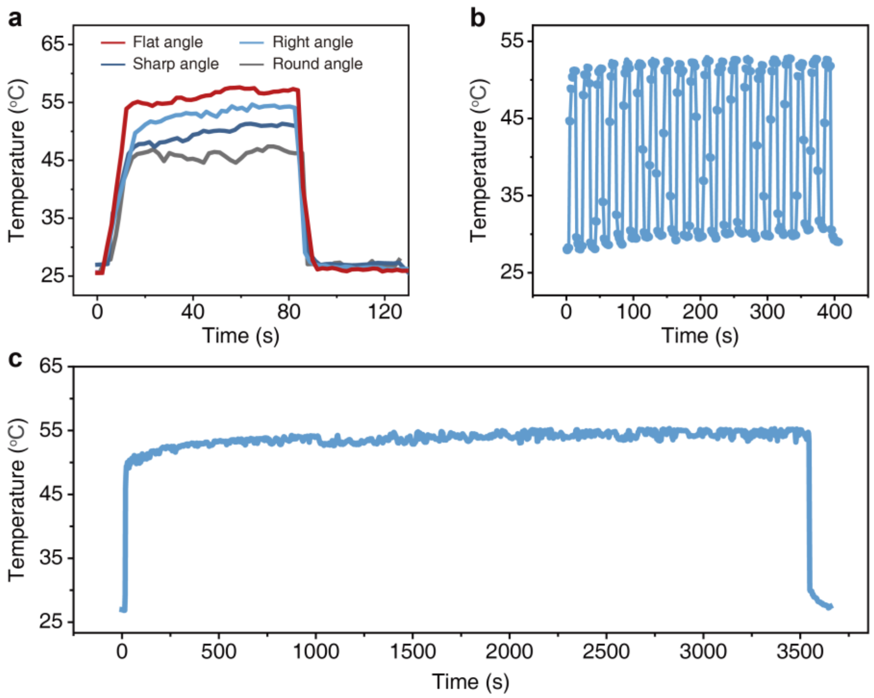

Figure S27. Quantitative Joule heating performance of 3D-printed CNTSP pathways. (a) Time-dependent surface temperature profiles for various print geometries (flat, right, sharp, and round angles) at an applied voltage of 10 V. (b) Cyclic heating–cooling temperature profiles for the right angle configuration. (c) Long-term stability test of a right-angle pathway at 10 V.

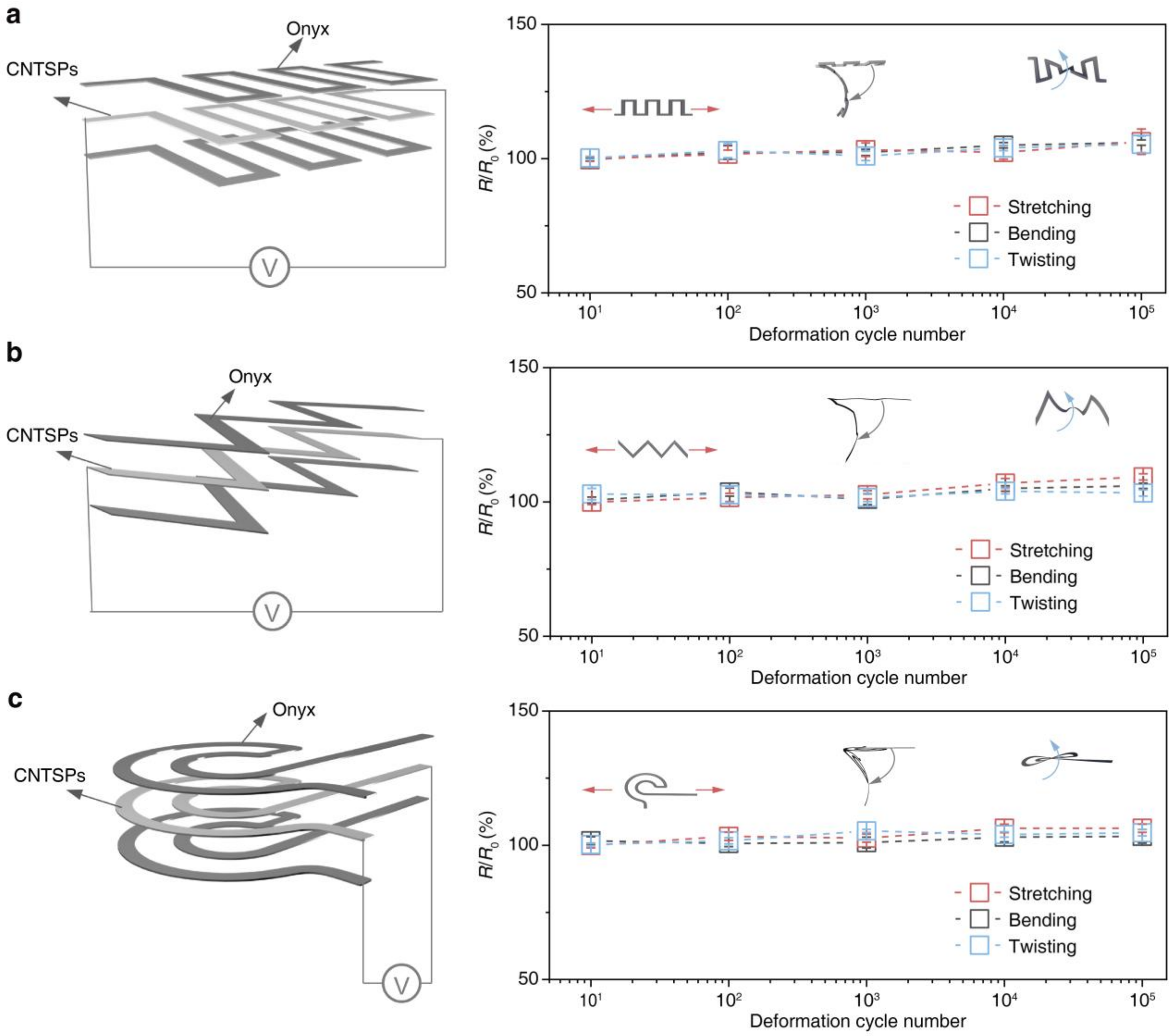

Figure S28. The cycle-dependence of the electrical resistance for different-shape circuits and the dependence of cycle number for rectangular a), triangular b), and c) semi-circular continuous circuits. This electrical resistance (R) is normalized to the resistance of the ribbon at room temperature before deformation (R0). After cyclic stretching the length of any of the structures for 100,000 cycles, the resistances increased by less than 10%. Also, bending any of the structures or twisting them for 100,000 cycles, the resistances increased by less than 10%. The bending was conducted by fixing one most-exterior lateral side of the structure and bending the opposite most-exterior lateral side by an angle of 60°, as shown in the inset. The twisting was conducted by fixing one most-exterior lateral side of the structure and twisting the opposite most-exterior lateral side by an angle of 30°, as shown in the inset.

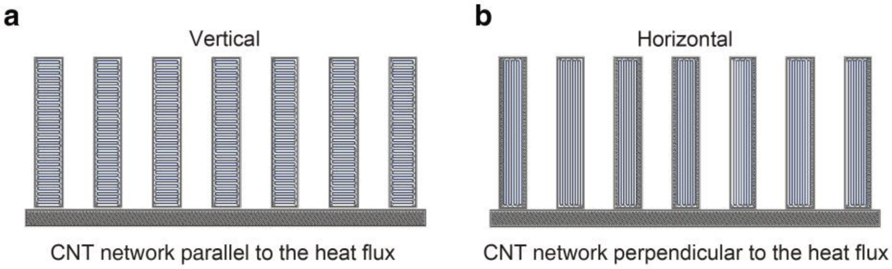

Figure S29. Images of the computer inputted structures for different CNTSPs printing paths. (a) 3D-printed vertical part within CNT network parallel to the heat flux. (b) 3D-printed vertical part within CNT network perpendicular to the heat flux.

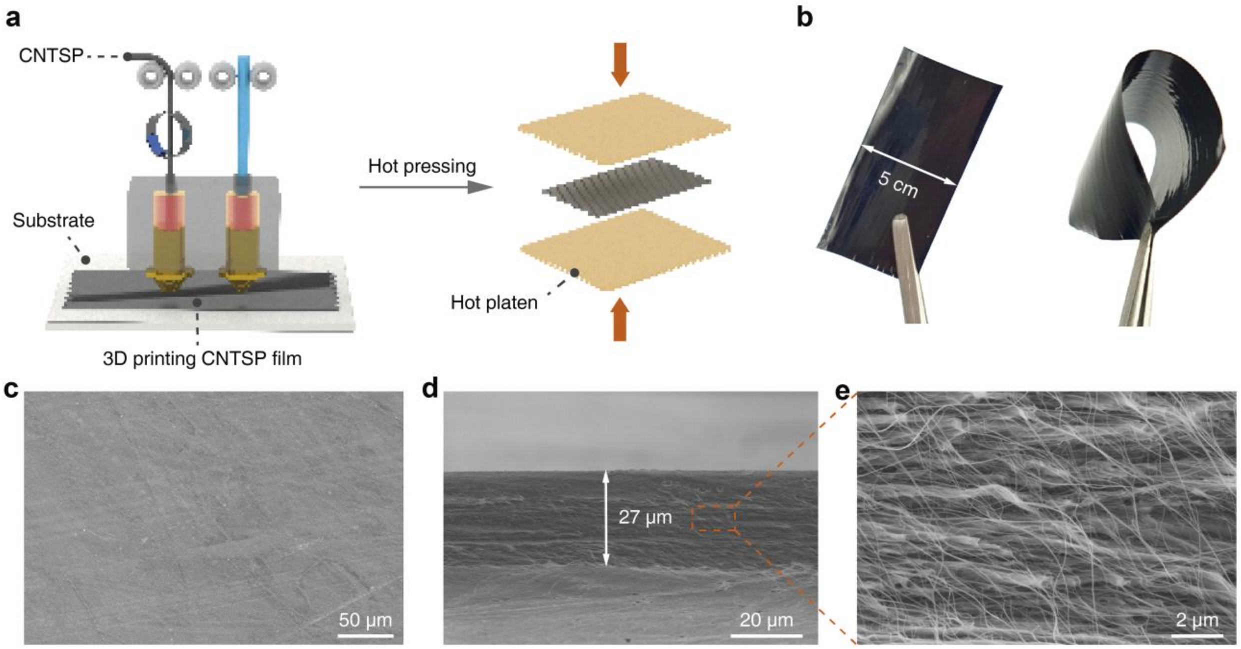

Figure S30. (a) Schematic diagram showing a printing of the CNT-direction oriented CNTSP film on a substrate from which the CNTSP film can be easily peeled, as well as the compression of the printing CNTSP film between two metal plates in order to provide the densification that increases CNT-direction thermal conductivity. (b) shows a photograph of the compressed CNTSP film. (c, d) show SEM images of the surface and the cross-section of the CNTSP film, respectively, and (e) shows a high-resolution image of the CNTSP film in the thickness direction.

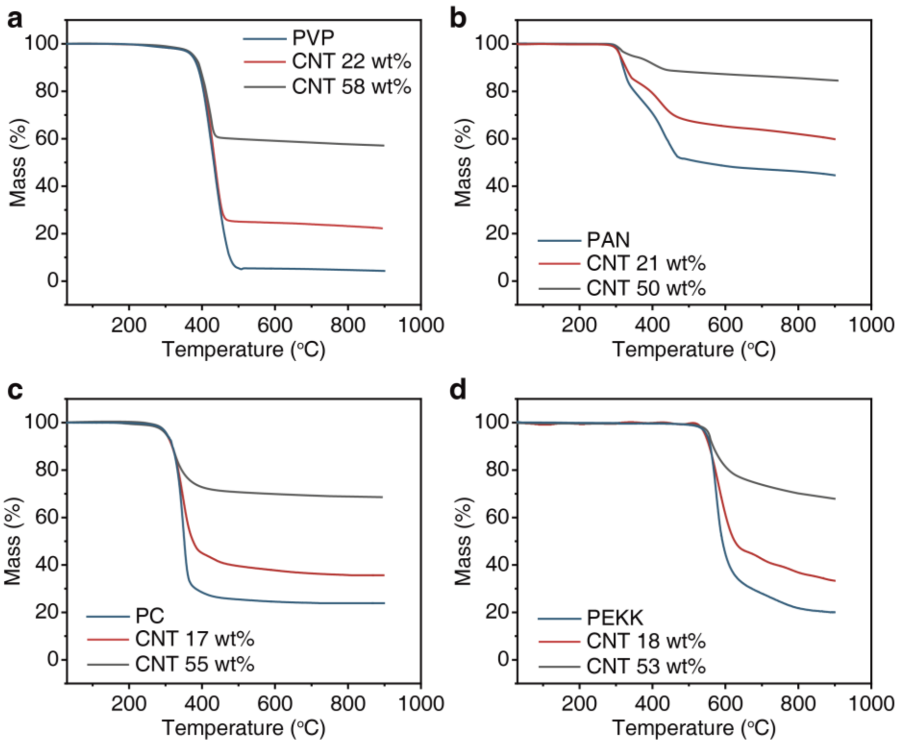

Figure S31. TGA curves of CNTSPs containing various polymers, as well as the TGA curves of these polymers.

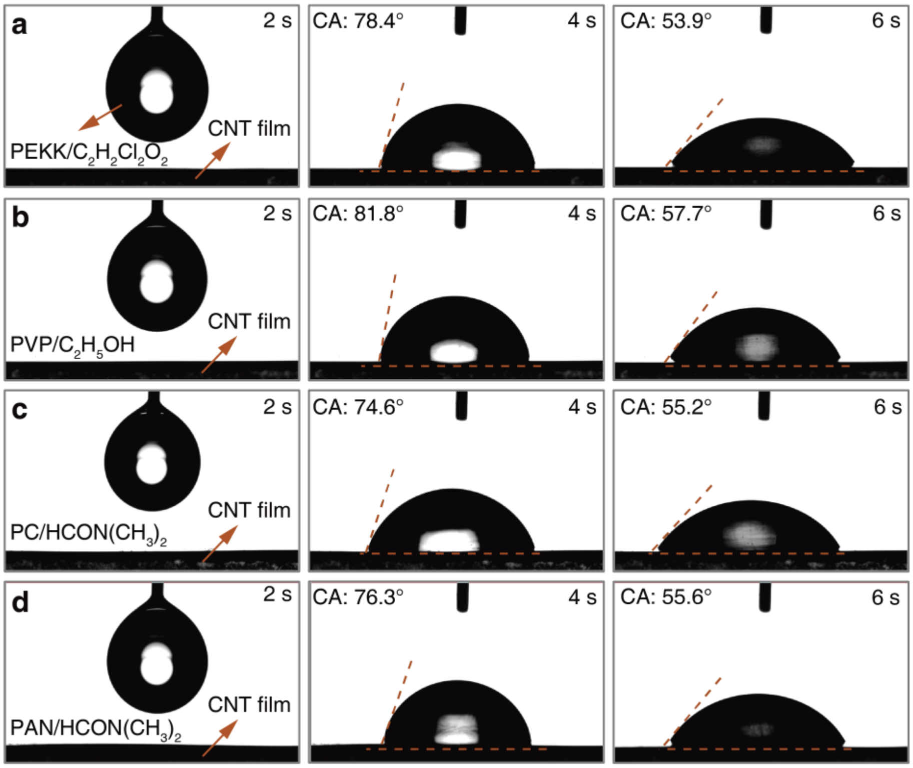

Figure S32. Photographs of the time dependence of contact angle measurements for various polymer solutions on CNT films. Time-dependent contact angle (CA) measurements of (a) 2 wt% PEKK/C2H2Cl2O2 solution, (b) 2 wt% PVP/C2H5OH solution, (c) 2 wt% PC/HCON(CH3)2 solution, and (d) 2 wt% PAN/HCON(CH3)2 solution on the surface of CNT films.

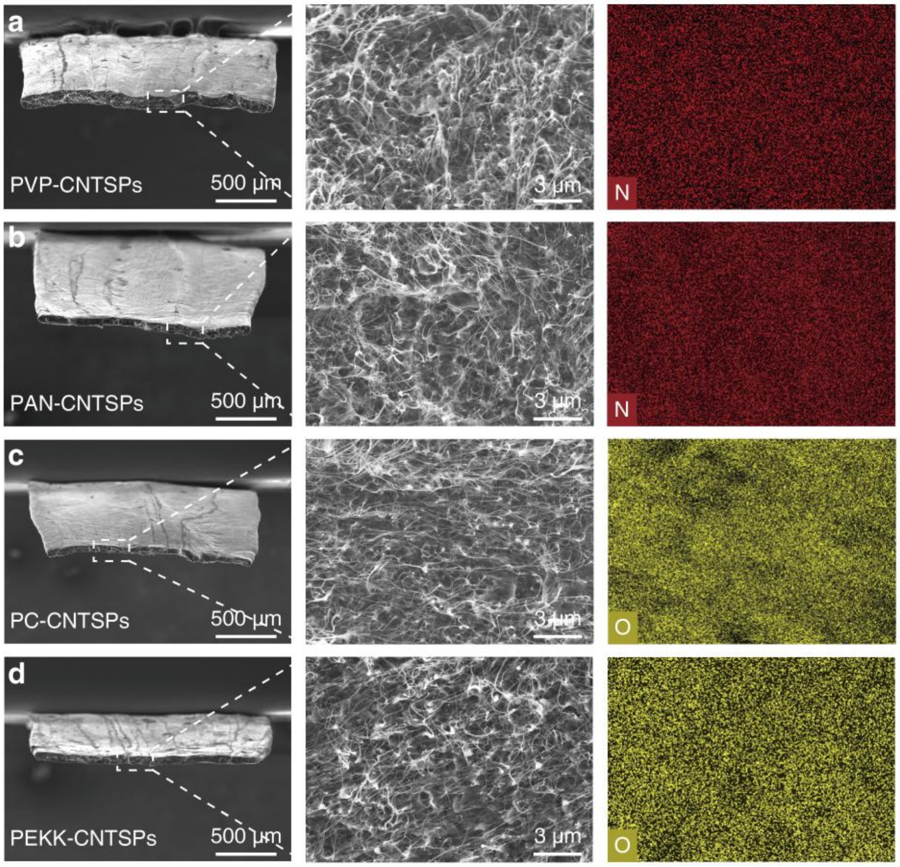

Figure S33. SEM images of CNTSP ribbons containing various polymers. Low-resolution (left), high-resolution cross-sectional SEM images (middle), and corresponding elemental mappings (right) of N (red) and O (yellow) atoms of (a) PVP-CNTSP, (b) PAN- CNTSP, (c) PC-CNTSP, and (d) PEKK-CNTSP ribbons.

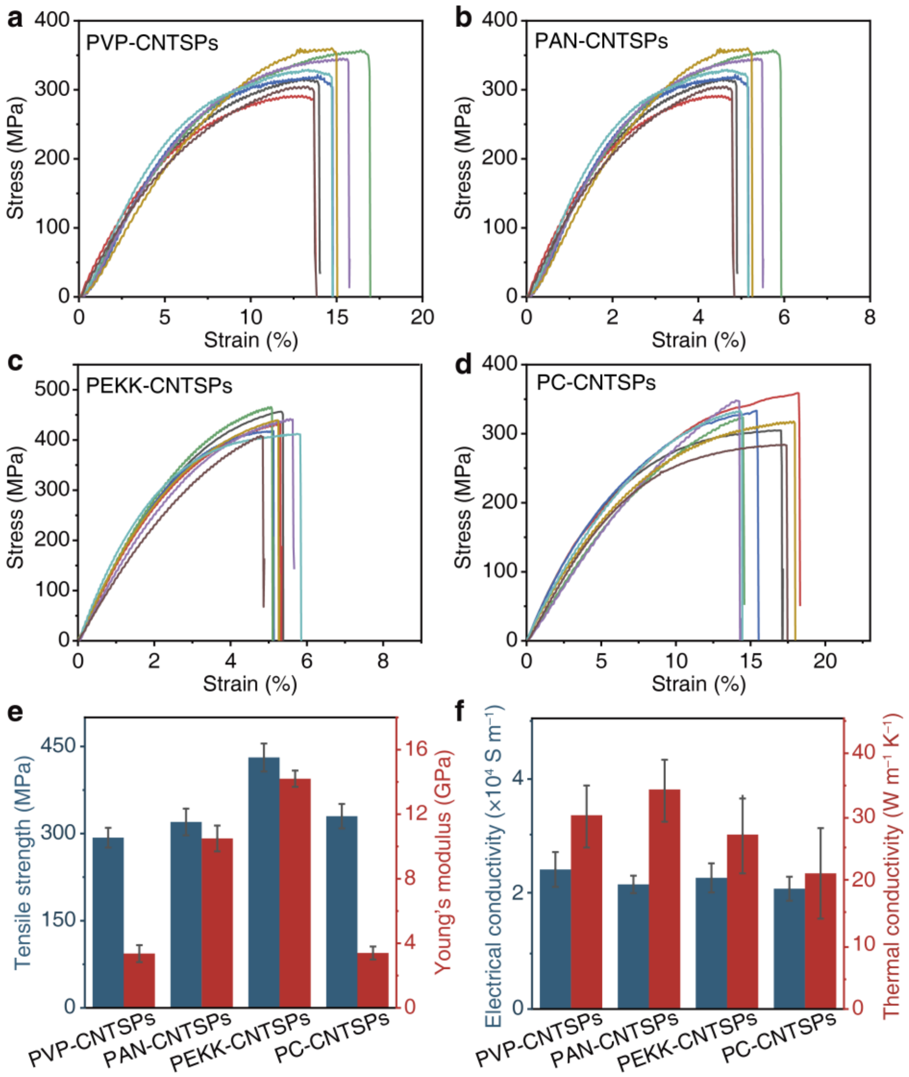

Figure S34. Mechanical, thermal, and electrical properties of PVP-CNTSP, PAN-CNTSP, PEKK-CNTSP, and PC-CNTSP ribbons with a low CNT mass fraction (<25 wt%). Stress-strain curves in the CNT orientation direction that result from 7-10 duplicate measurements on (a) PVP-CNTSP, (b) PAN-CNTSP, (c) PEKK-CNTSP, and (d) PC-CNTSP ribbons. (e) Tensile strength, Young's modulus, and f) electrical and thermal conductivity in the CNT orientation direction of PVP-CNTSP, PAN-CNTSP, PEKK-CNTSP, and PC-CNTSP ribbons. The indicated standard deviations were based on the 7-10 measurement results.

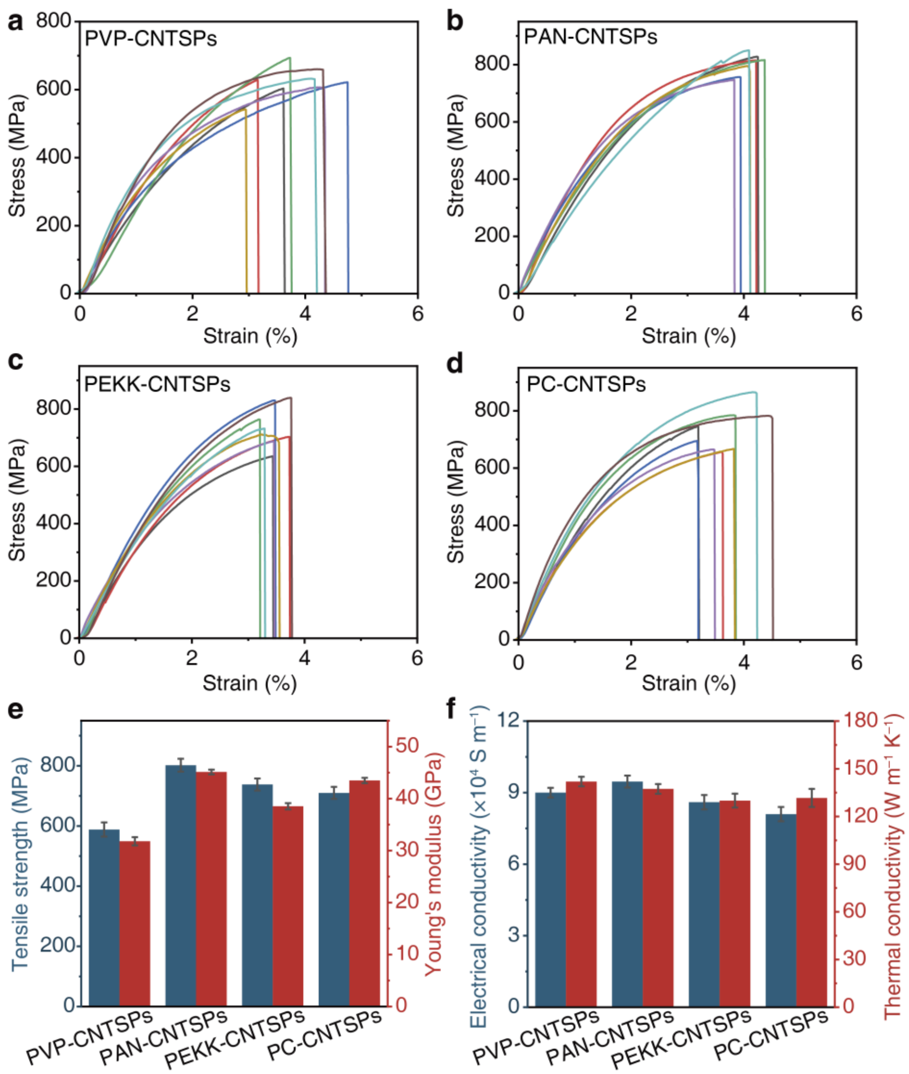

Figure S35. Mechanical, thermal, and electrical properties of PVP-CNTSP, PAN-CNTSP, PEKK-CNTSP, and PC-CNTSP ribbons with a high CNT mass fraction (>50 wt%). Stress-strain curves in the CNT orientation direction that result from 7-10 duplicate measurements on (a) PVP-CNTSP, (b) PAN-CNTSP, (c) PEKK-CNTSP, and (d) PC-CNTSP ribbons. (e) Tensile strength, Young's modulus, and (f) electrical and thermal conductivity in the CNT orientation direction of PVP-CNTSP, PAN-CNTSP, PEKK-CNTSP, and PC-CNTSP ribbons. The indicated standard deviations were based on the 7-10 measurement results.

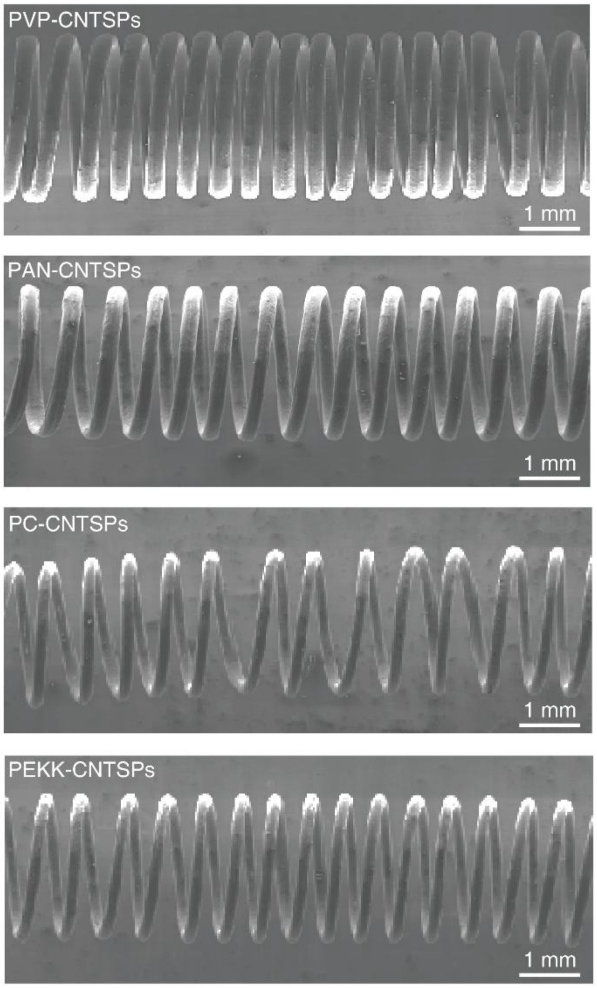

Figure S36. Photographs of coiled PVP-CNTSP, PAN-CNTSP, PC-CNTSP, and PEKK-CNTSP fibers.

Table S1. Comparison of thermal conductivities of various relevant materials.

Sample	Thermal conductivity (W m^{-1} K^{-1})	Ref.
PA6	0.13 ± 0.03	This work
CNT/PA6	0.45	S17
304-stainless steel	~15	35
Ultrahigh molecular weight polyethylene (UHMWPE) ultradrawn film along the drawn direction	62	8
Al-Si alloys (12 wt% Si)	121	36
CNTSPs (59 wt% CNT, κ_{max})	143 ± 5.8	This work

Table S2. Mass fractions and corresponding volume fractions of CNTs in CNTSPs.

Number	CNT mass fraction (wt%)	CNT volume fraction (vol%)
1	17	20
2	20	24
3	23	27
4	25	29
5	28	33
6	33	38
7	35	41
8	48	54
9	59	64
10	74	79

Table S3. Void microstructure distribution of CNTSP ribbons by nano-CT measurements.

Range (μm)	Mid-range (μm)	Volume (μm^3)	Percent volume in range (%)
0.20~0.60	0.4	3204	45.6
0.60~1.00	0.8	2885	41.1
1.00~1.40	1.2	743	10.6
1.40~1.80	1.6	169	2.4
1.80~2.20	2.0	18	0.3

Table S4. Void microstructure distribution of dry-state PA6/CNT networks by nano-CT measurements.

Range (μm)	Mid-range (μm)	Volume (μm^3)	Percent volume in range (%)
0.20~0.60	0.4	17398	23.0
0.60~1.00	0.8	28876	38.1
1.00~1.40	1.2	16147	21.3
1.40~1.80	1.6	9187	12.1
1.80~2.20	2.0	3254	4.3
2.20~2.60	2.4	745	1.0
2.60~3.00	2.8	186	0.2

Table S5. Mechanical, thermal, and electrical properties of CNTSP ribbons having different CNT mass fractions.

CNT mass fraction (wt%)	Tensile strength (MPa)	Young's modulus (GPa)	Electrical conductivity (S m^{-1})	κ_{max} (W m^{-1} K^{-1})
0	56 ± 11	0.7 ± 0.2	$\sim 10^{-13}$	0.13 ± 0.03
17	121 ± 18	2.23 ± 0.5	1.27×10^4	23 ± 6.9
20	226 ± 23	3.13 ± 0.5	1.80×10^4	29 ± 5.2
23	257 ± 20	4.21 ± 0.7	2.33×10^4	33 ± 4.6
25	320 ± 16	5.04 ± 0.4	3.04×10^4	42 ± 5.9
28	349 ± 19	5.56 ± 0.5	3.56×10^4	51 ± 7.1
33	380 ± 19	6.49 ± 0.5	4.26×10^4	67 ± 6.7
35	458 ± 25	7.18 ± 0.6	4.60×10^4	78 ± 7.3
48	551 ± 24	14.01 ± 0.9	6.70×10^4	115 ± 5.3
59	663 ± 18	18.12 ± 0.6	8.60×10^4	143 ± 5.8
74	780 ± 21	23.04 ± 0.8	10.10×10^4	176 ± 5.5

Table S6. Thermal anisotropy of CNTSP ribbons having different CNT mass fractions, where the thermal conductivity in the CNT orientation direction is κmax and through-plane thermal conductivity is κmin.

CNT mass fraction (wt%)	κ_{max} (W m^{-1} K^{-1})	κ_{min} (W m^{-1} K^{-1})	ρ ($\kappa_{max}/\kappa_{min}$)
0	0.13 ± 0.03	0.1 ± 0.04	1.3
17	23 ± 6.9	0.36 ± 0.01	63.8
20	29 ± 5.2	0.41 ± 0.02	70.7
23	33 ± 4.6	0.44 ± 0.01	75.0
25	42 ± 5.9	0.49 ± 0.06	85.7
28	51 ± 7.1	0.54 ± 0.04	94.4
33	67 ± 6.7	0.62 ± 0.02	108.0
35	78 ± 7.3	0.69 ± 0.04	113.0
48	115 ± 5.3	0.94 ± 0.04	112.3
59	143 ± 5.8	1.16 ± 0.01	123.2
74	176 ± 5.5	1.31 ± 0.02	134.3

Table S7. Comparison of thermal and electrical conductivities of various polymers reinforced by different nanofillers. The thermal and electrical conductivity are those measured in the highest respective conductivity direction.

Polymer	Filler	Filler content	Thermal conductivity ($W\ m^{-1}\ K^{-1}$)	Electrical conductivity ($S\ m^{-1}$)	Ref.
PP	CNT	20 wt%	~0.75	~100	S15
HDPE	CNT/BN	3 wt%/25 wt%	2.8	~10	S16
PA6	CNT	1 wt%	0.45	20	S17
PMMA	CF	30 wt%	0.3	~1.2	S18
PDMS	CF	18 wt%	3	100	S19
Epoxy	RGO	1 wt%	0.288	0.152	S20
PMMA	GF	0.4 wt%	0.27	0.035	S21
PMMA	GA	2.5 vol%	0.7	0.895	S22
PLA	GNs	9.08 vol%	3.22	0.3	S23
PVA	GNPs/BN	30 wt%/20 vol%	1.26	10^{-3}	S24
POM	CNT	40 wt%	1.95	3484	S25
PVA	GN	10 wt%	13.8	10	S26
CNF	RGO	50 wt%	7.3	4057.3	S27
PVDF	GNP	6 wt%	2.23	0.2	S28
PEKK	GNPs/CNT	19.84 vol%	6.27	524	S29
PS	Graphene	0.957 vol%	0.175	20.5	S30
PP	CNT/Graphene	2 wt%/13.01 wt%	0.82	1.45	S31
PVDF	GNP/CNT	2 wt%/20 wt%	0.69	2.13	S32

Note: Polypropylene (PP); high-density polyethylene (HDPE); polyamide 6 (PA6); polymethyl methacrylate (PMMA); polydimethylsiloxane (PDMS); epoxy resin (Epoxy); polylactic acid (PLA); polyvinyl alcohol (PVA); polyoxymethylene (acetal or polyacetal) (POM); cellulose nanofiber (CNF); polyvinylidene fluoride (PVDF); polyetherketoneketone (PEKK); polystyrene (PS).

Table S8. Comparison of tensile strength, thermal and electrical conductivities of various polymers reinforced by different nanofillers. The thermal and electrical conductivity are those measured in the highest respective conductivity direction.

Polymer	Filler	Filler content	Thermal conductivity (W m^{-1} K^{-1})	Electrical conductivity (S m^{-1})	Tensile strength (MPa)	Ref.
TPU	graphene	45 wt%	12.25	/	/	S33
LLDPE	graphene	3.51 vol%	3.11	~7	/	S34
PLA	graphene	45 wt%	3.82	/	/	S35
PLA	graphene	30 wt%	3.93	288.4	/	S36
TPU	BN	40 wt%	2.56	/	/	S37
TPU	BN	60 wt%	6.45	/	/	S38
LCP	BN	20 wt%	3.96	/	32.64	S39
PLA	BN	50 wt%	3.04	/	~32	S41
TPU	rGO/BN	35 wt%/22.02 vol%	2.61	/	/	S42
PA	graphite	10 wt%	5.5	/	/	S43
PC	SCF	10 vol%	~0.21	/	/	S44
ABS	SCF	2.65 vol%	0.20331	/	/	S45
PLA	CNT	20 wt%	0.575	/	/	S46
PPS	CNT	0.9 wt%	0.264	/	64	S47
ABS	CNT	8 wt%	0.14	/	/	S48
PA	SCF	30 wt%	1.8	/	~113	S49
PLA	graphene	45 wt%	3.82	/	19.3	S50
ABS	BN	35 wt%	1.45	/	~113	S51
PLA	graphene	5 wt%	~1	~1	20.9	S52
PU	CNT	1 wt%	16.5	10	/	S53
TPU	BN	30 wt%	1.8	/	~50	S54
PLA	graphene	9.08 vol%	3.22	78 S/m	/	S55

Note: Polyamide (PA); polylactic acid (PLA); TPU (Thermoplastic Polyurethane); LLDPE (Linear Low-Density Polyethylene); LCP (Liquid Crystal Polymer); PC (Polycarbonate); ABS (Acrylonitrile Butadiene Styrene); PPS (Polyphenylene Sulfide); PU (Polyurethane).

Table S9. Parameters in our rule-of-mixtures for different properties.

Property	P_c	P_{nyl}	α
Young's modulus (GPa)	46.1	0.7	1.76
Electric conductivity (10^4 S m^{-1})	17.5	0	1.32
Thermal conductivity (W m^{-1} K^{-1})	313.2	0.3	1.42

Table S10. Comparison of thermal conductivities along different directions for various 3D-printable plastics.

Polymer	Filler	Filler content	κ_{max} (W m^{-1} K^{-1})	κ_{min} (W m^{-1} K^{-1})	ρ ($\kappa_{max}/\kappa_{min}$)	Ref.
TPU	graphene	45 wt%	12.25	1.57	7.08	S33
LLDPE	graphene	3.51 vol%	3.11	1.02	3.04	S34
PLA	graphene	45 wt%	3.82	1.36	2.80	S35
PLA	graphene	30 wt%	3.93	0.76	5.17	S36
TPU	BN	40 wt%	2.56	0.91	2.81	S37
TPU	BN	60 wt%	6.45	~1.3	4.96	S38
LCP	BN	20 wt%	3.96	0.4	9.90	S39
TPU	BN	30 wt%	1.8	1.3	1.38	S40
PLA	BN	50 wt%	3.04	0.8	3.80	S41
TPU	rGO/BN	35 wt%/22.02 vol%	2.61	~0.53	4.92	S42
PA	graphite	10 wt%	5.5	~0.75	7.30	S43
PC	SCF	10 vol%	~0.21	~0.16	1.31	S44
ABS	SCF	2.65 vol%	0.20331	0.22171	1.09	S45
PLA	CNT	20 wt%	0.575	0.218	2.63	S46
PPS	CNT	0.9 wt%	0.264	0.228	1.15	S47
ABS	CNT	8 wt%	0.14	0.14	1	S48

Note: Polyamide (PA); polylactic acid (PLA); TPU (Thermoplastic Polyurethane); LLDPE (Linear Low-Density Polyethylene); LCP (Liquid Crystal Polymer); PC (Polycarbonate); ABS (Acrylonitrile Butadiene Styrene); PPS (Polyphenylene Sulfide).

Table S11. Mechanical, thermal, and electrical properties of PVP-CNTSP, PAN-CNTSP, PEKK-CNTSP, PC-CNTSP ribbons, and PA6 ribbons. The thermal and electrical conductivity are in the CNT orientation direction for the CNTSP ribbons and in the highest conductivity direction for PA6 ribbons.

Material	CNT mass fraction (wt%)	Tensile strength (MPa)	Young's modulus (GPa)	κ_{max} (W m^{-1} K^{-1})	Electrical conductivity (S m^{-1})
PVP	0	45 ± 10	3.7 ± 0.3	0.17 ± 0.02	$\sim 10^{-10}$
PVP-CNTSP	22	293 ± 17	3.8 ± 0.7	30 ± 6	2.40×10^4
PVP-CNTSP	58	586 ± 26	30.8 ± 0.6	142 ± 4	9.00×10^4
PAN	0	75 ± 11	3.2 ± 0.4	0.20 ± 0.01	$\sim 10^{-12}$
PAN-CNTSP	21	320 ± 23	10.5 ± 0.8	34 ± 4.7	2.13×10^4
PAN-CNTSP	50	803 ± 17	45.1 ± 0.3	138 ± 2.9	9.50×10^4
PEKK	0	113 ± 9	4.1 ± 0.2	0.23 ± 0.02	$\sim 10^{-13}$
PEKK-CNTSP	18	431 ± 24	14.2 ± 0.5	27 ± 4.7	2.27×10^4
PEKK-CNTSP	53	738 ± 20	38.4 ± 0.4	132± 3.1	8.60×10^4
PC	0	63 ± 14	2.2 ± 0.1	0.21 ± 0.03	$\sim 10^{-15}$
PC-CNTSP	17	330 ± 21	3.7 ± 0.6	21 ± 5.2	2.01×10^4
PC-CNTSP	55	710 ± 19	43.5± 0.3	130 ± 6.2	8.10×10^4
PA6	0	56 ± 11	0.7 ± 0.2	0.13 ± 0.03	$\sim 10^{-13}$

Supplementary References

1. Jeong HD, Kim SG and Choi GM *et al*. Theoretical and experimental investigation of the wet-spinning process for mechanically strong carbon nanotube fibers. *Chem Eng J* 2021; **412**: 128650.
2. Lu L, Yi W and Zhang D. 3ω method for specific heat and thermal conductivity measurements. *Rev Sci Instrum* 2001; **72**: 2996–3003.
3. Qiu L, Guo P and Yang X *et al*. Electro curing of oriented bismaleimide between aligned carbon nanotubes for high mechanical and thermal performances. *Carbon* 2019; **145**: 650–657.
4. Laurent C, Flahaut E and Peigney A. The weight and density of carbon nanotubes versus the number of walls and diameter. *Carbon* 2010; **48**: 2989–99.
5. Hourahine B, Aradi B and Blum V *et al*. DFTB+, A software package for efficient approximate density functional theory based atomistic simulations. *J Chem Phys* 2020; **152**: 124101.
6. Grimme S, Antony J and Ehrlich S *et al*. A consistent and accurate ab initio parametrization of density functional dispersion correction (DFT-D) for the 94 elements H-Pu. *J Chem Phys* 2010; **132**: 154104.
7. Grimme S, Ehrlich S and Goerigk L. Effect of the damping function in dispersion corrected density functional theory. *J Comput Chem* 2011; **32**: 1456–65.
8. Gaus M, Goez A and Elstner M. *et al*. Parametrization and benchmark of DFTB3 for organic molecules. *J Chem Theory Comput* 2013; **9**: 338–54.
9. Gaus M, Lu X and Elstner M *et al*. Parameterization of DFTB3/3OB for sulfur and phosphorus for chemical and biological applications. *J Chem Theory Comput* 2014; **10**: 1518–37.
10. Lu X, Gaus M and Elstner M *et al*. Parametrization of DFTB3/3OB for magnesium and zinc for chemical and biological applications. *J Phys Chem B* 2015; **119**: 1062–82.
11. Plimpton S. Fast parallel algorithms for short-range molecular dynamics. *J Comput Phys* 1995; **117**: 1–19.
12. Sun H, Mumby SJ and Maple JR *et al*. An ab initio CFF93 all-atom force field for polycarbonates. *J Am Chem Soc* 1994; **116**: 2978–87.
13. Lordi V, Yao N. Molecular mechanics of binding in carbon-nanotube-polymer composites. *J Mater Res* 2000; **15**: 2770–9.
14. Hockney RW and Eastwood JW. *Computer Simulation using Particles*. Taylor & Francis, 1989.
15. Wu K, Xue Y and Yang W *et al.* Largely enhanced thermal and electrical conductivity via constructing double percolated filler network in polypropylene/expanded graphit-Multi-wall carbon nanotubes ternary composites. *Compos Sci Technol* 2016; **130**: 28–35.
16. Che J, Jing M and Liu D *et al.* Largely enhanced thermal conductivity of HDPE/boron nitride/carbon nanotubes ternary composites via filler network-network synergy and orientation. *Compos Part A Appl Sci Manuf* 2018; **112**: 32–9.
17. Park M, Lee H and Jang J *et al.* Phenyl glycidyl ether as an effective noncovalent functionalization agent for multiwalled carbon nanotube reinforced polyamide 6 nanocomposite fibers. *Compos Sci Technol* 2019; **177**: 96–102.
18. Elimat ZM, Hussain WT and Zihlif AM. PAN-based carbon fibers/PMMA composites: Thermal, dielectric, and DC electrical properties. *J Mater Sci Mater Electron* 2012; **23**: 2117–22.
19. He X, Huang Y and Liu Y *et al*. Improved thermal conductivity of polydimethylsiloxane/short carbon fiber composites prepared by spatial confining forced network assembly. *J Mater Sci* 2018; **53**: 14299–310.

20. Osman A, Elhakeem A and Kaytbay S *et al*. electrical and mechanical properties of graphene/nano-alumina/epoxy composites. *Mater Chem Phys* 2021; **257**: 123809.
21. Zhang C, Li A and Zhao Y *et al*. Thermal, electrical and mechanical properties of graphene foam filled poly(methyl methacrylate) composite prepared by in situ polymerization. *Compos Part B Eng* 2018; **135**: 201–6.
22. Fan Z, Gong F and Nguyen ST *et al*. Advanced multifunctional graphene aerogel – Poly (methyl methacrylate) composites: Experiments and modeling. *Carbon* 2015; **81**: 396–404.
23. Shi S, Dai M and Tao X *et al*. 3D printed polylactic acid/graphene nanocomposites with tailored multifunctionality towards superior thermal management and high-efficient electromagnetic interference shielding. *Chem Eng J* 2022; **450**: 138248.
24. Xu W, Franklin R and Ravichandran D *et al*. Continuous nanoparticle patterning strategy in layer-structured nanocomposite fibers. *Adv Funct Mater* 2022; **32**: 2204731.
25. Li J, Wang Y and Gao T *et al*. Robust electromagnetic interference shielding, joule heating, thermal conductivity, and anti-dripping performances of polyoxymethylene with uniform distribution and high mass fraction of carbon-based nanofillers. *Compos Sci Technol* 2021; **206**: 108681.
26. Zhuang Y, Zheng K and Cao X *et al*. Flexible graphene nanocomposites with simultaneous highly anisotropic thermal and electrical conductivities prepared by engineered graphene with flat morphology. *ACS Nano* 2020; **14**: 11733–42.
27. Yang W, Zhao Z and Wu K *et al*. Ultrathin flexible reduced graphene oxide/cellulose nanofiber composite films with strongly anisotropic thermal conductivity and efficient electromagnetic interference shielding. *J Mater Chem C* 2017; **5**: 3748–56.
28. Lei Y, Bai Y and Shi Y *et al*. Composite nanoarchitectonics of poly(vinylidene fluoride)/graphene for thermal and electrical conductivity enhancement via constructing segregated network structure. *J Polym Res* 2022; **29**: 213.
29. Chen R, He Q and Li X *et al*. Significant enhancement of thermal conductivity in segregated(GnPs&MWCNTs)@Polybenzoxazine/(Polyether ether ketone) -based composites with excellent electromagnetic shielding. *Chem Eng J* 2022; **431**: 134049.
30. Zhao F, Zhang G and Zhao S *et al*. Fabrication of pristine graphene-based conductive polystyrene composites towards high performance and light-weight. *Compos Sci Technol* 2018; **159**: 232–9.
31. Hu S, Xu B and Zhao Y *et al*. Preparation of CNTs/PP@Gr composites with a segregated structure and enhanced electrical and thermal conductive properties by the Pickering emulsion method. *Compos Sci Technol* 2022; **222**: 109374.
32. Xiao Y, Wang W and Chen X *et al*. Hybrid network structure and thermal conductive properties in poly(vinylidene fluoride) composites based on carbon nanotubes and graphene nanoplatelets. *Compos Part A Appl Sci Manuf* 2016; **90**: 614–25.
33. Guo H, Zhao H and Niu H *et al*. Highly thermally conductive 3D printed graphene filled polymer composites for scalable thermal management applications. *ACS Nano* 2021; **15**: 6917−28.
34. Peng Z, Lv Q and Jing J *et al*. FDM-3D printing LLDPE/BN@GNPs composites with double network structures for high-efficiency thermal conductivity and electromagnetic interference shielding. *Compos Part B Eng* 2023; **251**: 110491.
35. Fang B, Zhang G and Zou F *et al*. 3D printing interconnected segregated composites to simultaneously enhance thermal conductivity and mechanical properties. *ACS Appl Polym Mater* 2024; **6**: 4904−911.

36. Ma T, Ma H and Ruan KP *et al*. Thermally conductive poly(lactic acid) composites with superior electromagnetic shielding performances via 3D printing technology. *Chin J Polym Sci* 2022; **40**: 248–55.
37. Liu J, Li W and Guo Y *et al*. Improved thermal conductivity of thermoplastic polyurethane via aligned boron nitride platelets assisted by 3D printing. *Compos Part A Appl Sci Manuf* 2019; **120**: 140–6.
38. Guo H, Niu H and Zhao H *et al*. Highly anisotropic thermal conductivity of three-dimensional printed boron nitride-filled thermoplastic polyurethane composites: effects of size, orientation, viscosity, and voids. *ACS Appl Mater Interfaces* 2022; **14**: 14568−78.
39. Luo F, Yang S and Yan P *et al*. Orientation behavior and thermal conductivity of liquid crystal polymer composites based on Three-Dimensional printing. *Compos Part A Appl Sci Manuf* 2022; **160**: 1070592.
40. Gao J, Hao M and Wang Y *et al*. 3D printing boron nitride nanosheets filled thermoplastic polyurethane composites with enhanced mechanical and thermal conductive properties. *Addit Manuf* 2022; **56**: 102897.
41. Li S, Fang B and Wang Y *et al*. Three-dimensional printing of boron nitride platelets/polylactic acid composites: achieving enhanced through plane thermal conductivity and decent mechanical properties. *Polym Compos* 2024; **46**: 4605–13.
42. Wang S, He H and Ye X *et al*. Design of rGO-BN hybrids for enhanced thermal management properties of polyurethane composites fabricated by 3D printing. *Compos Sci Technol* 2022; **227**: 109591.
43. Jia Y, He H and Geng Y *et al*. High through-plane thermal conductivity of polymer based product with vertical alignment of graphite flakes achieved via 3D printing. *Compos Sci Technol* 2017; **145**: 55-61.
44. Gupta A, Hasanov S and Fidan I. Thermal characterization of short carbon fiber reinforced high temperature polymer material produced using the fused filament fabrication process. *J Manuf Process* 2022; **80**: 515–28.
45. Shemelya C, Rosa ADL and Torrado AR *et al*. Anisotropy of thermal conductivity in 3D printed polymer matrix composites for space based cube satellites. *Addit Manuf* 2017; **16**: 186–96.
46. Zhang C, Deng K and Li X *et al*. Thermally conductive 3D-printed carbon-nanotube-filled polymer nanocomposites for scalable thermal management. *ACS Appl Nano Mater* 2023; **6**: 13400−8.
47. Pan S, Shen H and Zhang L. Effect of carbon nanotube on thermal, tribological and mechanical properties of 3D printing polyphenylene sulfide. *Addit Manuf* 2021; **47**: 102247.
48. Guadagno L, Aliberti F and Longo R *et al*. Electrical anisotropy controlled heating of acrylonitrile butadiene styrene 3D printed parts. *Mater Des* 2023; **225**: 111507.
49. Ramanathan A, Thippanna V and Kumar AS *et al*. Highly loaded carbon fiber filaments for 3D-printed composites. *J Polym Sci* 2024; **62**: 2670–82.
50. Fang B, Zhang G and Zou F *et al*. 3D printing interconnected segregated composites to simultaneously enhance thermal conductivity and mechanical properties. *ACS Appl Polym Mater* 2024; **6**: 4904−11.
51. Quill TJ, Smith MK and Zhou T *et al*. Thermal and mechanical properties of 3D printed boron nitride – ABS composites. *Appl Compos Mater* 2018; **25**: 1205–17.

52. Kotsilkova R, Ivanov E and Georgiev V *et al*. Essential nanostructure parameters to govern reinforcement and functionality of poly(lactic) acid nanocomposites with graphene and carbon nanotubes for 3D printing application. *Polymers* 2020; **12**: 1208.
53. Yuan S, Zheng Y and Chua C *et al*. Electrical and thermal conductivities of MWCNT/polymer composites fabricated by selective laser sintering. *Compos. Part A Appl Sci Manuf* 2018; **105**: 203–13.
54. Gao J, Hao M and Wang Y *et al*. 3D printing boron nitride nanosheets filled thermoplastic polyurethane composites with enhanced mechanical and thermal conductive properties. *Addit Manuf* 2022; **56**: 102897.
55. Shi S, Dai M and Tao X *et al*. 3D printed polylactic acid/graphene nanocomposites with tailored multifunctionality towards superior thermal management and high-efficient electromagnetic interference shielding. *Chem Eng J* 2022; **450**: 138248.
